# SNAI1 ablation alters integrin-mediated adhesion and endocytic fate

**DOI:** 10.1038/s41419-026-09179-x

**Published:** 2026-08-12

**Authors:** Chrysoula Tsirigoti, Mohamad Moustafa Ali, Anita Morén, Staffan Johansson, Michael J. Munson, Carl-Henrik Heldin, Aristidis Moustakas, Dorival Mendes Rodrigues-Junior

**Affiliations:** 1 Department of Medical Biochemistry and Microbiology, Science for Life Laboratory, Uppsala University, Uppsala, Sweden; 2 Advanced Drug Delivery, Pharmaceutical Sciences, BioPharmaceuticals R&D, AstraZeneca, Mölndal, Sweden

**Keywords:** Breast cancer, Membrane trafficking

## Abstract

Transcription factor SNAI1 guides plasticity and invasiveness in cancer. Using a complete *SNAI1* knockout in mesenchymal, triple-negative breast cancer cells, unbiased genome-wide transcriptomic analysis revealed a marked under-expression of integrin-based adhesion and endocytic components. Utilizing this knockout cell model, complementary breast cancer cell models and functional screening of multiple differentially expressed genes, we found that the pioneering transcription factor FOXA1, whose expression is repressed by SNAI1, associates with several key mediators of the cellular phenotype. FOXA1 represses the small GTPase ARF6 and its exchange factor PSD4. In addition, some of the integrin and matrix metalloproteinase genes are regulated by the transcriptional FOXA1 signal. Accordingly, *SNAI1* knockout cells presented poor adhesion to collagen type I or fibronectin, formed defective invadopodia and focal adhesions with weakened FAK/SRC signaling. *SNAI1* knockout cells performed ineffective receptor-mediated internalization, including nanoparticle and extracellular vesicle (EV) uptake, exhibited reduced lysosomal content, lacked multivesicular bodies enriched in intraluminal vesicles and showed decreased EV secretion. Gain-of-function experiments demonstrated that SNAI1 has an impact on the PSD4/ARF6 signaling module, using FOXA1 as an intermediate factor to regulate EV release by tumor cells. We propose that the SNAI1-FOXA1 transcriptional mechanism operates at the level of membrane and vesicular trafficking control, which interlinks cell plasticity, adhesion and invasiveness through the extracellular environment, with the associated process of EV secretion.

## Introduction

Triple-negative breast cancers (TNBC) lack estrogen and progesterone receptors, while also lacking HER2 or occasionally expressing distinct epidermal growth factor family receptors [1]. Mesenchymal (claudin-low) TNBCs express reduced epithelial and enhanced mesenchymal proteins, hallmarks of epithelial-mesenchymal transition (EMT), which promotes invasiveness [2]. EMT is driven by growth factor pathways, such as transforming growth factor β (TGF-β) signaling via SMAD and protein kinases, which induce expression of EMT-related transcription factors (EMT-TFs), including SNAI1 [2]. In breast cancer (BRCA), SNAI1 represses epithelial junctional genes (e.g., E-cadherin, occludin) and epithelial pioneering factors, such as FOXA1, preventing the emergence of luminal epithelial cells [3–5], while inducing the expression of mesenchymal junctional genes (e.g., N-cadherin) [6], and glycolytic enzymes (e.g., fructose-1,6-bisphosphatase) [7]. Notably, SNAI1 depletion in TNBC or ovarian cancer triggers mesenchymal-to-epithelial transition, favoring cell-cell adhesion, reducing invasive potential and promoting luminal trans-differentiation [5, 8–10].

Upon TGF-β-induced EMT, cancer cells remodel their extracellular matrix (ECM), form invadopodia, and secrete extracellular vesicles (EVs) that mediate intercellular communication within the tumor microenvironment [2, 11, 12]. In breast epithelial EMT, TGF-β signaling transcriptionally induces integrin β3, which pairs with TGF-β receptors and reinforces their signaling. This positive feedback promotes activation of the SRC and focal adhesion (FAK) tyrosine kinases [13], promoting cell migration on fibronectin and collagen type I [14]. Following EMT induction, SNAI1 enhances secretion of the ECM-degrading proteases cathepsin-B around invadopodia and of metalloproteinases (MMPs) in colorectal and hepatocellular carcinoma cells [15, 16]. Mesenchymal cell migration depends on the organization of actin into linear microfilaments at the leading edge (lamellipodia), which are enriched in complexes between surface receptors and ECM adhesion proteins [17, 18]. Forward movement is enabled as actomyosin‑generated tension is released through the disassembly of focal adhesions at the rear of the cell [19]. Endocytic vesicular trafficking has a crucial role in establishing this front-rear polarity, driving both the speed and persistence of cell adhesion and migration [20, 21].

While the molecular circuitry that regulates and interconnects endocytic trafficking to adhesion is complex, two regulators stand out beyond the integrins: the ADP-ribosylating factor 6 (ARF6) and its guanine exchange factor pleckstrin-and-Sec7 domain-containing-4 (PSD4) [22]. PSD4 is downregulated in TNBCs and mesenchymal tumor cells and antagonizes TGF-β-induced EMT [22]. By activating ARF6, PSD4 promotes receptor endocytosis, including E-cadherin internalization during EMT [23, 24], and regulates RAC1/actin cytoskeleton dynamics to support mesenchymal migration [25, 26]. ARF6 also cooperates with additional partners, such as GGA3 and Rabaptin5, which form an endosomal sorting complex that facilitates early recycling of integrin β1, driving cell migration [27]. Thus, the PSD4/ARF6 signaling unit regulates alternative fates in breast cells, ranging from luminal morphogenesis to pro-invasive cell behavior.

In a different context than cell migration affected by PSD4/ARF6, EMT increases endosomal trafficking to alter cell polarity and enhance motility of lung adenocarcinoma cells [28]. Endosomal trafficking via endosomal sorting complex required for transport (ESCRT) and RAB family small GTPases, among other functions, also contributes to the biogenesis of small EVs (e.g., exosomes) that can be additionally regulated by tetraspanins (e.g., CD9, CD63 and CD81), lipid metabolism and ARF6 [29]. In this context, ARF6 associates with phospholipase D2 to control the budding of intraluminal vesicles into multivesicular bodies (MVBs) [30]. EVs secreted by TNBCs carry a diverse set of molecular cargo, contributing to pre-metastatic niche formation and to the development of immune surveillance resistance [29, 31]. Once secreted, EV surface ligands interact with recipient cell receptors, fuse with the plasma membrane, or are taken up by endocytosis/phagocytosis, impacting cellular responses [32]. Nevertheless, whether transcriptional programs of EMT can impact endocytic trafficking and EV biology remains sparsely studied. Thus, motivated by the striking phenotypic plasticity detected in TNBC MDA-MB-231 cells lacking *SNAI1* expression [5], our transcriptomic analyses uncovered a coordinated collapse of cell‑matrix interaction programs. Specifically, we detected loss of integrin expression and adhesion signatures, accompanied by reduced PSD4/ARF6 levels, further implicating defects in endocytosis and EV secretion. These findings point to a SNAI1‑dependent regulatory module that safeguards trafficking and adhesion networks. Here, we dissect the components of this gene circuitry operating downstream of SNAI1.

## Materials and methods

### Cell culture, reagents and treatments

Human MDA-MB-231 (HTB-26: basal-TNBC), ZR-75-1 (CRL-1500: luminal BRCA) and MCF7 (HTB-22: luminal BRCA) cells were obtained from ATCC (LGC Standards, UK), and cultured in Dulbecco’s modified Eagle’s medium (DMEM; Thermo Fisher Scientific, Stockholm, Sweden) or Roswell Park Memorial Institute 1640 medium (RPMI; Thermo Fisher Scientific, Stockholm, Sweden), supplemented with 10% fetal bovine serum (FBS; Biowest, Almeco A/S, Esbjerg, Denmark), 100 U/ml penicillin and 100 μg/ml streptomycin (Sigma-Aldrich AB, Stockholm, Sweden). All cells were kept in a humidified incubator at 37 °C and 5% CO_2_. MDA-MB-231 *SNAI1*-KO cells, clones CS16 and CS19, were generated by the CRISPR/Cas9 system and cultured as previously described [5]. Cells were analyzed and found free of mycoplasma and authenticated using STR analysis. Cells were starved overnight in serum-free DMEM and treated for different time periods with recombinant human TGF-β1 (PreproTech EC Ltd, London, UK) at a final concentration of 5 ng/ml, as indicated.

### RNA-seq, gene set enrichment analyses and peak annotation

RNA extraction from biological triplicate samples, representing either stimulated or unstimulated conditions, was performed using the ReliaPrep™ RNA Cell Miniprep System (Promega, Madison, WI, USA). Following RNA quality assessment, 500 ng of total RNA was subjected to library preparation using the TruSeq stranded total RNA Gold library preparation kit with RiboZero Gold treatment and unique dual indexes according to the manufacturer’s instructions (Protocol # 1000000040499, Illumina Inc., San Diego, CA, USA). Paired-end sequencing was carried out with v1.5 sequencing chemistry on a NovaSeq-6000 platform using S4 flow cells (Illumina Inc., USA). The RNA-seq raw files were processed as previously described [5]. Differential gene expression analysis was performed utilizing the DESeq2 Bioconductor package in R, considering any transcript with log2 fold change ±1 and false discovery rate (FDR) < 0.05. Data visualization was done in RStudio v1.4.1717 with R v4.0.5. Gene set enrichment analysis (GSEA) was carried out with the GSEA tool and the molecular signature database MSigDBv6 [33]. ChIP-seq peaks contained in BED files were annotated using the annotatePeaks.pl program within the HOMER v4.11 suite.

### Immunoblotting and antibodies

Cellular proteins were extracted and immunoblotted as previously described [5]. The membranes were blocked with 5% BSA (Saveen Werner, Limhamn, Sweden) and then incubated with primary antibodies and horseradish peroxidase-conjugated anti-mouse or anti-rabbit secondary antibodies as listed in Supplementary Table S1, followed by enhanced chemiluminescence assays using the Millipore kit (Merck/Millipore, Stockholm, Sweden). All uncropped (original) immunoblots are shown as a supplemental file.

### cDNA synthesis and reverse transcription quantitative PCR

Total RNA was extracted using the Nucleospin RNA plus Kit (Macherey-Nagel, AH Diagnostics, Solna, Sweden). The concentration of RNA was measured on a NanoDrop 2000 (Thermo Fisher Scientific, Stockholm, Sweden) and 1 μg of RNA was used for reverse transcription using the iScript cDNA synthesis kit (Bio-Rad Laboratories Inc., Sundbyberg, Sweden), according to the manufacturer’s protocol. Real-time qPCR was performed on a Bio-Rad CFX96 cycler (Bio-Rad Laboratories Inc., Sundbyberg, Sweden) using the qPCRBIO SyGreen 2× Master Mix (PCR Biosystems, London, UK). The expression levels of the target genes were normalized to the expression levels of the reference gene *GAPDH* and relative normalized expression was calculated based on the ΔΔC_t_ method. The results were plotted in graphs as average values of the relative normalized expression. The qPCR primers are listed in Supplementary Table S2.

### Chromatin immunoprecipitation

For each chromatin immunoprecipitation (ChIP) reaction, 5 × 10^6^ cells were utilized, either unstimulated or stimulated with TGF-β1 and ChIP was performed as previously described [34]. Quantitative PCR was performed, as mentioned above, using the primers listed in Supplementary Table S2 and *GAPDH* as a negative control.

### ProteinAtlas, TCGA data analysis and overall survival analysis

mRNA expression data for tumor cell lines were obtained from the Human Protein Atlas (https://www.proteinatlas.org). The log2-transformed RNA expression values of the investigated genes were obtained from the cBioportal platform [35]. Overall survival (OS) of basal BRCA patients was predicted using the KM plotter database [36]. Samples were stratified and compared using auto-select cut-off, and the *p*-value was calculated by the log-rank test.

### siRNA and plasmid transfections

Using distinct human-specific siRNA oligonucleotides (ON-TARGETplus Non-targeting Control Pool (D-001810-10), ON-TARGETplus Human FOXA1 set of four individual siRNAs (LQ-010319-00-0002, Dharmacon/VWR, Stockholm, Sweden), 20 nM of each siRNA was transfected using Lipofectamine RNAiMAX according to the manufacturer’s instructions (Thermo Fisher Scientific, Stockholm, Sweden). Cells collected 48 h after transfection were validated for knockdown efficiency by RT-qPCR and were seeded for additional experiments.

*SNAI1*-KO cells were transfected using X-tremeGENE 360 transfection reagent (Merck/Millipore, Stockholm, Sweden) with plasmids pcDNA3.1-HA or Flag empty vectors, pcDNA3.1-SNAI1-HA, -SNAI1-FLAG, constitutively active ALK5-HA (TGF-β type I receptor) described in [37], or pcCtrl-GFP and pcARF6-GFP (#67394, Addgene, Watertown, MA, USA). After 48 h, cells were harvested for RNA-, protein-, or cell-based assays.

### Extracellular matrix-mediated cell adhesion

Cells were allowed to attach for 60 min to 96-well plates coated with the ECM proteins fibronectin (40 μg/ml, isolated from blood plasma) or collagen type I (0.05 mg/ml, PureCol, Advanced BioMatrix, Inc., San Diego, CA, USA). Pluronic F-127 (10 mg/ml, Sigma-Aldrich AB, Stockholm, Sweden) was used to block any uncoated surface of the coated wells prior to cell seeding and as a negative control. To confirm the role of integrin β1 in promoting cell adhesion, MDA-MB-231 WT cells were pre-treated with 5 µg/mL IgG or integrin β1 blocking monoclonal antibody (mAb; clone P5D2; MAB1959; Millipore-Sigma, Stockholm, Sweden) for 1 h prior to transfer into 96-well plates coated with fibronectin or collagen type I. Adherent cells were stained with crystal violet in MetOH, solubilized with MetOH at room temperature for 30 min and quantified at OD 570 nm in an Enspire cell counter (PerkinElmer Sverige AB, Upplands Väsby, Sweden).

### Cell-surface protein biotinylation

Following overnight starvation, sub-confluent cells were washed with PBS and treated for 30 min on ice with 0.5 mg/ml of membrane-impermeable and cleavable EZ-link^TM^ Sulfo-NHS-SS biotin (21331, Thermo Fisher Scientific, Stockholm, Sweden). Cells were transferred to 37 °C to initiate endocytosis and washed with ice-cold PBS. Unincorporated biotin was quenched with 0.1 M glycine for 10 min and cells were washed with ice-cold PBS before staining with Alexa Fluor 488-streptavidin conjugate (Thermo Fisher Scientific, Stockholm, Sweden) at a dilution of 1:1000 in PBS for 1 h at room temperature. The nuclei were stained with 4´, 6-diamidino-2-phenylindole (DAPI; Sigma-Aldrich AB, Stockholm, Sweden) at a dilution of 1:1000 in PBS for 15 min at room temperature. The coverslips were mounted with Fluoromount-G (Southern Biotech, AH Diagnostics, Solna, Sweden) and examined by a Nikon Eclipse 90i fluorescence microscope (Nikon Corp., Tokyo, Japan). Five to six random pictures were taken with a 20× objective at the same exposure time.

MDA-MB-231 cells were cultured in medium containing 1% FBS overnight before stimulation with 5 ng/ml TGF-β1 for 15 or 30 min, followed by washing twice in ice-cold PBS, pH 7.3, and gentle shaking incubation with 0.5 mg/ml sulfo-NHS-SS-biotin (Thermo Fisher Scientific/Pierce) in PBS, pH 8.0 for 1 h at 4 °C. Labeling reactions were stopped by incubation in ice-cold 50 mM Tris, pH 8.0 for 10 min. Cells were then washed in PBS, pH 7.3, and lysed in the same reagent described above, under immunoblotting. Biotinylated proteins in the cell lysates were incubated with neutravidin agarose beads (Thermo Fisher Scientific/Pierce) for 1 h at 4 °C; the beads were then washed four times in ice-cold lysis buffer, whereafter protein eluates were obtained by boiling for 5 min in sodium dodecyl sulphate (SDS) sample buffer prior to polyacrylamide gel electrophoresis (SDS-PAGE) and immunoblotting. All uncropped (original) immunoblots are shown as a supplemental file.

### Transferrin receptor recycling assays

Recycling of transferrin receptor (TfR) was analyzed by direct fluorescence microscopy as previously described [27]. Briefly, MDA-MB-231 cells were placed on ice and incubated with 5 µg/mL Alexa Fluor™ 488–conjugated transferrin (Trf; T13342, Thermo Fisher Scientific, Stockholm, Sweden) for 45 min. After two PBS washes, cells were returned to 37 °C to allow Trf internalization. Following 15 min of internalization, cells were placed back on ice and incubated for 45 min in medium containing 10% FBS, 25 µg/mL unlabeled holo‑Trf (T4132, Merck, Stockholm, Sweden), and anti–Alexa Fluor® 488 antibody (#A11094, Thermo Fisher Scientific, Stockholm, Sweden) to quench remaining surface‑bound fluorescence. Cells were then washed twice with PBS and incubated in complete medium at 37 °C. At the indicated time points, cells were fixed in 4% w/v paraformaldehyde in PBS for 15 min at room temperature.

### Fluorescent gelatin degradation assay

Glass-bottom 4-well chamber slides were coated with 50 μg/ml poly-D-lysine (Thermo Fisher Scientific, Stockholm, Sweden) for 30 min at room temperature, washed with PBS and fixed with 0.5% w/v glutaraldehyde for 10 min. Wells were washed with PBS three times and incubated with 0.2% w/v Oregon green 488-labeled gelatin (D12054, Thermo Fisher Scientific, Stockholm, Sweden) in 2% w/v sucrose in PBS for 10 min at room temperature. Wells were washed with PBS and incubated with 0.1 M glycine for 30 min, washed three times with PBS and incubated with complete medium for 30 min. Cells were seeded at 1 × 10^5^ per well and incubated at 37 °C for 2 days. Cells were then washed with PBS and fixed with 3.7% w/v formaldehyde for 15 min at room temperature and processed for labeling with phalloidin and DAPI prior to fluorescence microscopy as described under immunofluorescence. Five to six random pictures were taken with a 20× objective at the same exposure time. For the quantification of gelatin degradation, the total area of the degraded matrix, as measured by the ImageJ software, was divided by the number of cells present in the total area of each picture.

### Generation of mCherry-GAL9 reporter cell lines

Stable MDA-MB-231 WT, CS16 and CS19 *SNAI1*-KO cells expressing mCherry-GAL9 were generated by co-transfection of an *AAVS1-* targeting zinc-finger nuclease and the reporter plasmid encoding mCherry-GAL9 under the *E1α* promoter, allowing for nuclease-mediated targeted integration through non-homologous end joining [38]. Upon seeding 2 × 10^5^ cells per well in a 12-well plate and overnight incubation at 37 °C, cells were transfected with the mCherry-GAL9 reporter (*AAVS1* locus at 1:9 ratio) using FuGENE HD transfection reagent (Promega, Madison, WI, USA) according to the manufacturer’s instructions. Transfected mCherry-GAL9 cells were sorted into similar expression pools by flow cytometry and expanded for further use.

### Fluorescence-activated cell sorting (FACS)

MDA-MB-231 WT, CS16 and CS19 *SNAI1*-KO cells, transfected or not with a mCherry-GAL9 reporter, were expanded to 80% confluency. Cells were washed with PBS, detached with TrypLE Express for 10 min at 37 °C and resuspended in FACS buffer (5% FBS in PBS) upon filtration in FACS tubes using a 35 μm strainer (Falcon, Saveen & Werner AB, Limhamn, Sweden). Cell sorting was performed on a SONY SH800 Cell Sorter. For each cell type, non-transfected cells were used in order to set up the voltage of the forward scatter (FSC) and the side scatter (SSC), as well as to adjust the autofluorescence level in the mCherry channel. Cellular debris was excluded by plotting the FSC- area vs the SSC- area with all events and gating for living cells. One pool of mCherry-GAL9-positive cells was sorted in a tube and the cells were grown in the presence of penicillin (100 µg/ml)-and streptomycin (100 µg/ml) for a week. Cells were then grown in antibiotic-free culture medium and used for further experiments.

### High throughput live mCherry-GAL9-reporter imaging

mCherry-GAL9-reporter cell lines were seeded into a 384-well Cell-Carrier Ultra plate (6007558, PerkinElmer Sverige AB, Upplands Väsby, Sweden) and incubated in complete media overnight prior to treatment. Hoechst 33342 was added to the cell culture medium at 0.5 μg/ml a minimum of 30 min prior to start of the imaging experiment. The reporter cell lines were then exposed to lipid nanoparticles (LNPs) formulated by microfluidic mixing of Cy5-labeled mRNA with lipids, using the ionizable lipid Dlin-MC3-DMA (MC3) to enable delivery, as previously described [38]. Hoechst, lipid nanoparticles and PBS, the latter serving as a control, were dispensed into the cell culture medium using an Echo 655T acoustic dispenser (Labcyte, Indianapolis, USA). Immediately after dosing, the cell plate was moved to the microscope and imaged. Live-cell imaging was carried out at a CV8000 (Yokogawa, Musashino, Japan) confocal microscope within a humidified chamber maintained at 37 °C and 5% CO_2_. Images were obtained using a 20× objective and laser wavelengths of 405 nm (BP445/45 nm), 561 nm (BP600/37) or 640 nm (BP676/29) for Hoechst, mCherry and Cy5, respectively. For time course experiments, the same fields of view were imaged over time. Images were processed and analyzed using Signals Image Artist (SImA) v1.3.77 image analysis software. Cells were identified using nuclear Hoechst and cell boundary mCherry-GAL9 signals. Within each cell region of interest, spot populations were quantified using a “Find Spots” building block within SImA that identifies punctate structures with relative intensities higher than local cellular background intensity. Data were exported and analyzed in Spotfire Analyst (Tibco, v11.4.0) where Cy5 and mCherry spots were normalized against nuclear Hoechst (cell number) before export and plotting in Excel (Microsoft, v2511).

### Lysosomal tracing

Lysosomes were quantified using LysoTracker Deep Red (L12492, Thermo Fisher Scientific, Stockholm, Sweden), a cell-permeable, fluorescent dye that stains acidic cell compartments. After seeding 5 × 10^4^ cells per well in 24-well plates and culturing for 48 h, followed by incubation with 50 nM of the LysoTracker probe for 60 min at 37 °C, the cells were fixed in 3.7% w/v formaldehyde and subjected to flow cytometric analysis, in which 1 × 10^4^ events were counted (Acuri, BD Biosciences, Stockholm, Sweden). The flow cytometry profiles were displayed as histograms, and results were plotted according to the median of positive cells.

### EV isolation and characterization

FBS was depleted from EVs by centrifugation at 150,000 × *g* for 18 h followed by filtration through 2.0 μm filters. Cells, at 50% confluency, were cultured in DMEM supplemented with 10% EV-depleted FBS for 3 days. Conditioned media (CM) were collected, the corresponding number of cells was measured and EVs were enriched by ultracentrifugation. Briefly, CM was first centrifuged at 200 × *g* for 5 min; the supernatant was then centrifuged a second time at 16,000 × *g* for 30 min, followed by centrifugation at 120,000 × *g* for 90 min. The pellet was resuspended in PBS and the ultracentrifugation fraction (UCF) was collected and stored at −80 °C. Alternatively, the CM was collected and the corresponding number of cells was measured; the CM was then centrifuged at 200 × *g* for 5 min to clear cell debris, re-centrifuged at 10,000 × *g* for 5 min. After filtering (0.2 μm), the vesicular secretome fraction (VSF) was concentrated 20× on a 100 kDa Ultra-15 Centrifugal Filter (Amicon; Merck, Stockholm, Sweden) exposed to 4000 × *g* centrifugation. To enrich CD81-positive EV subpopulations, 150 µL of VSF were incubated with 40 µL of anti-CD81 magnetic Dynabeads™ (#10616D, Thermo Fisher Scientific, Stockholm, Sweden) for 60 min at 37 °C with gentle agitation. Following incubation, bead-bound CD81-positive EVs were immobilized using a magnetic rack and washed with PBS. The enriched CD81-positive EVs were lysed in RIPA buffer (50 mM Tris-HCl pH 7.5, 150 mM NaCl, 1% NP-40, 0.5% sodium deoxycholate and 0.1% SDS) and lysates were resolved by SDS-PAGE. The particle size and number were quantified by nanoparticle tracking analysis (NTA) in a LM10 NanoSight (Malvern Panalytical, Malvern, UK). Transmission electron microscopy (TEM) was performed to visualize EVs present in the UCF or VSF by negative staining, as previously described [2, 11, 12].

### Immuno- and direct fluorescence microscopy

Cells were fixed in 3.7% w/v formaldehyde for 15 min at room temperature, incubated with 0.1 M glycine for 30 min and permeabilized in 0.5% v/v Triton X-100 for 10 min at room temperature. Then, the samples were blocked in 5% w/v BSA in PBS for 1 h at room temperature and incubated with the primary antibodies (Supplementary Table S1) in 1% BSA in PBS overnight at 4 °C. After washing, the samples were incubated with Alexa-Fluor-488 or Alexa-Fluor-546 secondary antibodies (Invitrogen, Thermo Fisher Scientific, Stockholm, Sweden) at a dilution of 1:500 in PBS for 1 h at room temperature. For EV uptake assays, the VSF enriched from MDA-MB-231 WT cells was labeled with PKH26 red (#MIDI26-1KT, Sigma-Aldrich, Stockholm, Sweden), as previously described [11, 12].

Invadopodial formation was assessed with cortactin staining. Briefly, 1 × 10^5^ MDA-MB-231 WT and *SNAI1*-KO cells were seeded on 4-well glass chamber slides coated with 0.03 mg/ml collagen type I (PureCol, Advanced BioMatrix, Inc., San Diego, CA, USA) for 2 days at 37 °C and cells were then treated as described above. TRITC-labeled phalloidin (Sigma-Aldrich AB, Stockholm, Sweden) was used at a dilution of 1:500 in PBS for 1 h at room temperature. The nuclei were stained with DAPI at a dilution 1:1000 in PBS for 15 min at room temperature, and samples were analyzed microscopically as described above.

### Cellular transmission electron microscopy

In order to track endocytosis and identify MVBs, 1 × 10^5^ cells per well were seeded in a 6-well plate and 24 h later subjected to overnight serum starvation. Cells were washed with PBS and incubated with 10% FBS in DMEM, supplemented with 0.5% 10 nm gold-labeled BSA (25487, Electron Microscopy Sciences, PA, USA) for 3 h. The cells were fixed in 2.5% w/v glutaraldehyde (Ted Pella Inc., Redding, CA, USA)/1% w/v paraformaldehyde (Merck/Millipore, Stockholm, Sweden) in PIPES buffer pH 7.4, washed in 0.1 M phosphate buffer, pH 7.4 (PB) for 10 min prior to 30 min incubation in 1% w/v osmium tetroxide (TAAB Laboratories Equipment Ltd, Berks, UK) in 0.1 M PB. After a further wash in 0.1 M PB, cells were dehydrated in graded (70–99.9%) alcohols. The wells were then filled with a 1:1 mixture of Epon resin (Ted Pella Inc., Redding, CA, USA) and 99.9% EtOH for 1 h, followed by 100% resin and left overnight. The next day, the resin was replaced with freshly prepared resin and then polymerized at 60 °C for 48 h. Ultra-thin sections (60-70 nm) of the cell layer were produced in a Leica UC7 Ultramicrotome (Leica Geosystems AG, Wetzler, Germany) and placed on grids. The grids were contrasted in 5% uranyl acetate and 3% Reynolds lead citrate for 10 and 2 min, respectively, prior to examination on a transmission electron microscope (FEI Tecnai G2, JEOL, Tokyo, Japan) operated at 80 kV. Images were captured using an ORIUS SC200 CCD camera and processed with Gatan Digital Micrograph software (Gatan Inc./Blue Scientific, Pleasanton, CA, USA).

### Active ARF6 pulldown assay

Cells at 80% confluency were washed with PBS and lysed on ice according to the manufacturer’s instructions (16121, Thermo Fisher Scientific, Stockholm, Sweden). As a control, 1/10 of the total cell lysate was saved as input, and the rest of the cleared lysates were incubated with resin carrying 100 μg of N-terminally glutathione S-transferase-tagged human Golgi-associated, γ-adaptin ear-containing, ARF-binding protein 3 (GST-GGA3), which binds with high affinity to ARF6-GTP, for 1 h at 4 °C under gentle rotation. The samples were then washed, heated at 95 °C in sample buffer for 5 min and equal amounts of protein were subjected to SDS-PAGE.

### Data analysis and statistics

Results express the mean values of at least two independent experiments (biological repeats), as explained in the figure legends. Error bars represent the standard error of the mean (SEM), unless otherwise noted. Two-group comparisons were performed using two-tailed unpaired student’s *t*-test. Multiple comparisons were performed using one-way or two-way analysis of variance (ANOVA), followed by multiple paired comparisons conducted by means of the Tukey’s post-test method when applicable. Data analysis was performed using GraphPad Prism version 10.4 (GraphPad Software, San Diego, CA). Statistical significance is represented by *p*-values *p ≤ 0.05, **p ≤ 0.01, ***p ≤ 0.001, ****p ≤ 0.0001. The number of replicates is indicated in every figure legend. After determining the efficiency of each cell-based assay, the number of technical and biological repeats was defined, and the appropriate statistical method was selected based on sample content and variation within each dataset included for comparison. Error bars represent the standard error of the mean SEM and occasionally SD. The variance was similar between the groups that have been compared. Accordingly, the statistical method reported in the figure legends was chosen.

## Results

### *SNAI1* knockout impairs cell-matrix adhesion and endocytic gene profiles in TNBC-basal cells

**Fig. 1:**
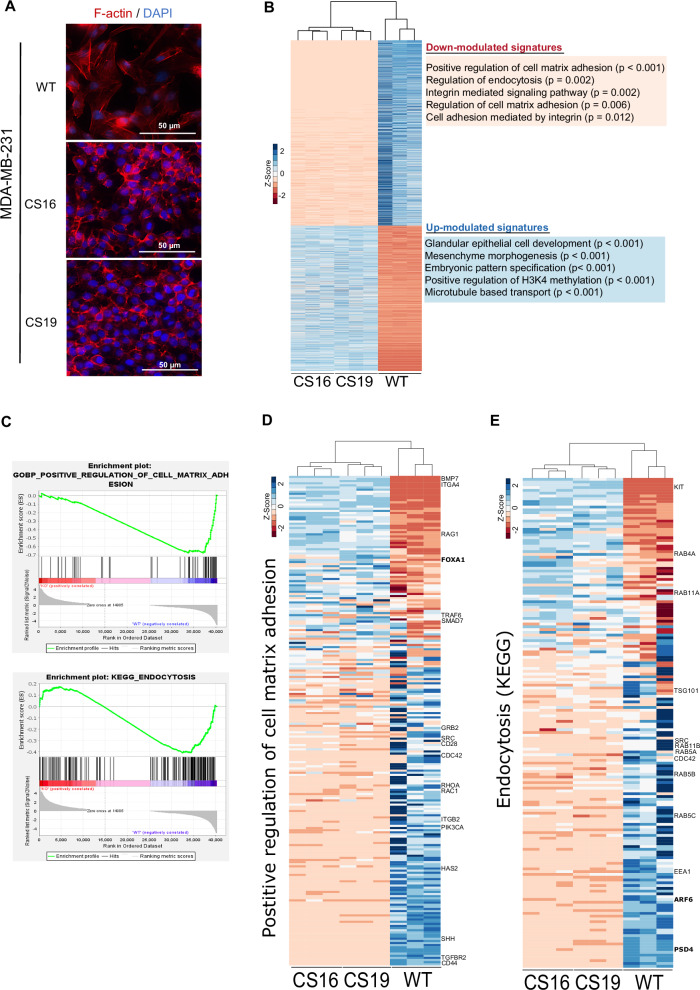
Defective endocytosis and cell matrix adhesion gene profiles in *SNAI1* knockout cells. **A** Representative F-actin (red) staining images of MDA-MB-231 WT and *SNAI1*-KO cells. Nuclei are stained in blue with DAPI. Scale bars, 50 μm. **B** Heatmap demonstrating differential gene expression (up- and down-regulated genes), highlighting highly enriched molecular signatures based on the KEGG database. **C** Enrichment plots of the significantly enriched signatures of regulation of extracellular matrix adhesion and endocytosis in *SNAI1*-KO cells, with normalized enriched scores of −1.3 and −1.67, respectively. Heatmaps showing expression levels of differentially expressed genes involved in the regulation of cell matrix adhesion (GOBP) (**D**) and endocytosis (KEGG) (**E**). In **B**, **D** and **E**, the color-coded scale represents normalized counts per million values of WT and *SNAI1*-KO cells. Key genes related to the phenotypes and analyzed in this study are marked.

Previously, we stably ablated the *SNAI1* gene in mesenchymal and invasive TNBC-basal BRCA cells that highly express *SNAI1* [5, 9]. Upon *SNAI1* deletion (MDA-MB-231 CS16, CS19 clones), cells acquired an intermediate epithelio-mesenchymal phenotype (Fig. 1A) with concomitant suppression of the EMT-TF ZEB1, of the TGF-β signaling pathway and invasiveness, and gain of cell differentiation plasticity and proliferative potential [5]. Differential gene expression analysis revealed that cell-matrix adhesive and endocytic pathways were among the most significantly downregulated molecular signatures in *SNAI1*-KO cells (Fig. 1B, C), whereas glandular epithelial cell development, morphogenesis and embryonic patterning were upregulated, reflecting the central conclusion of our previous study [5]. Among the differentially expressed genes, we identified: (a) upregulation of antagonistic (*BMP7*) or negative (*SMAD7*) regulators of TGF-β signaling or mediators of non-SMAD signaling (*TRAF6*), and downregulation of central signaling mediators (*TGFBR2*), coreceptors (*CD44*) and downstream effectors (*HAS2*) of TGF-β signaling; (b) downregulation of focal adhesion, cytoskeletal organization, motility and invasiveness signaling genes (*ITGB2*, *RHOA*, *RAC1*, *CDC42*, *SRC*; however, *ITGA4* was upregulated); (c) upregulation of the pioneering transcription factor *FOXA1*, as previously established [5]; (d) upregulation of recycling effectors, the RAB GTPases (*RAB4A*, *RAB11A*), and downregulation of endocytic GTPases, ESCRT effectors and accessory genes (*RAB5A*, *B*, *C*, *TSG101*, *EEA1*); and (e) downregulation of bifunctional regulators of luminal morphogenesis and invasiveness (*PSD4*, *ARF6*) (Fig. 1D, E).

Among these profound changes in gene expression profile, we decided to focus on integrins because of their key role in cell-ECM adhesion, and on *PSD4* and *ARF6* because of their dual functions in luminal morphogenesis and cancer cell invasiveness and possible linking functions between endocytic and adhesive mechanisms. As an effort to connect this work to our recent analysis of *FOXA1* as a downstream gene regulated by SNAI1, we also decided to analyze the impact of FOXA1 while confirming the relative inactivation of TGF-β signaling in these new investigations of TNBC cell biology. Accordingly, we validated the reduction of mRNA and protein levels of PSD4 and ARF6 in *SNAI1*-KO cells (Supplementary Fig. 1A–C). By inspecting the expression levels of *FOXA1*, PSD4 and *ARF6* in a panel of breast, lung and ovarian cancer cell lines (https://www.proteinatlas.org), we could attest relative variation of expression for each gene among the cell lines, and relative concordance between mRNA (Supplementary Fig. 1B) and protein (Supplementary Fig. 1C) levels in some of these cell lines.

### FOXA1 associates with regulatory sequences and represses *PSD4* and *ARF6* expression

**Fig. 2:**
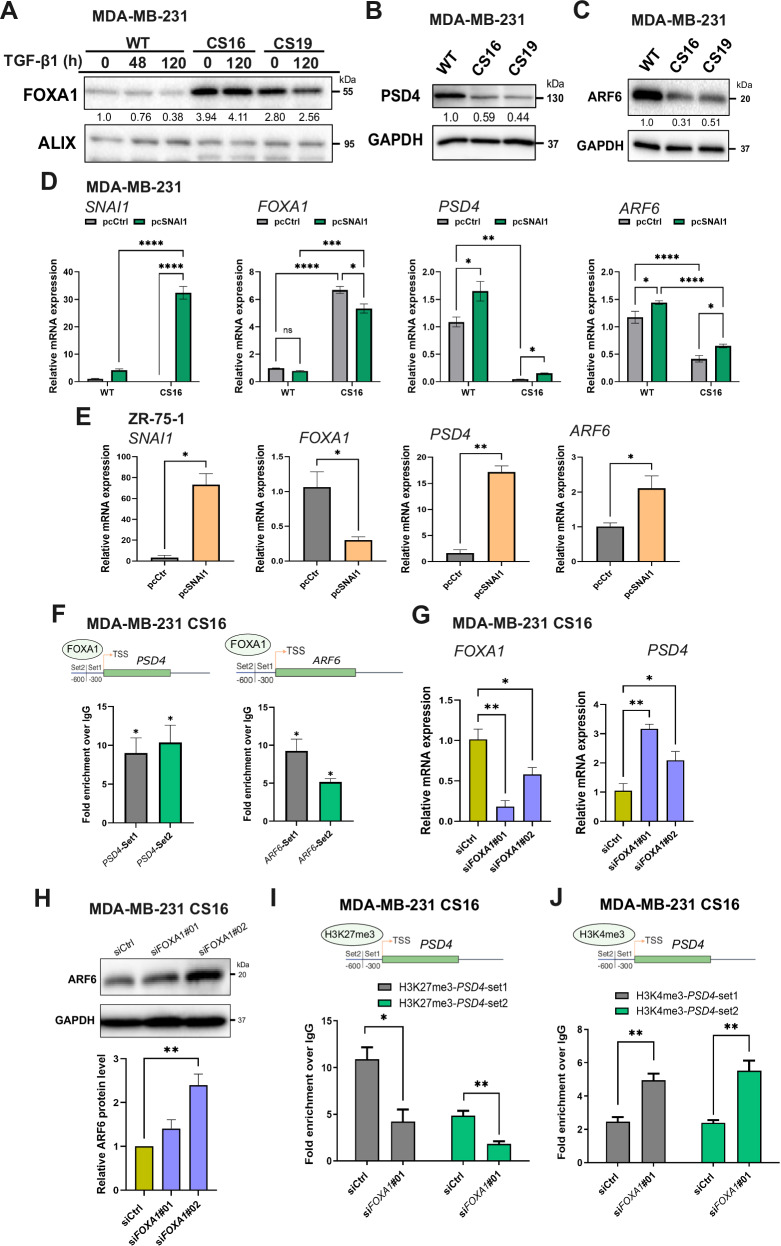
Loss of SNAI1 releases FOXA1 expression, a repressor of the *PSD4* and *ARF6* genes. **A** Representative immunoblot and average densitometric analysis of three biological replicates along with molecular mass markers in kDa showing protein expression levels of FOXA1 in MDA-MB-231 WT and *SNAI1*-KO cells, stimulated with 5 ng/ml TGF-β1 for the indicated time periods. ALIX serves as a loading control. Protein expression levels of PSD4 (**B**) and ARF6 (**C**) in MDA-MB-231 WT and *SNAI1*-KO cells and average densitometric analysis. GAPDH serves as a loading control. Immunoblots are representative of three biological replicates along with molecular mass markers in kDa. RT-qPCR analysis of *SNAI1*, *FOXA1*, *PSD4* and *ARF6* mRNA levels in MDA-MB-231 WT (**D**) and ZR-75-1 (**E**) cells transiently transfected with empty vector (Ctrl) or vectors expressing FLAG-tagged form of SNAI1 (pcSNAI1). Values represent fold-change of mRNA expression normalized to *GAPDH* and expressed relative to the level in MDA-MB-231 WT cells. **F** ChIP-qPCR analysis of FOXA1 binding in two different regions (600 and 300 bp) upstream of the *PSD4* (left) and *ARF6* (right) transcription starting sites in CS16 *SNAI1*-KO cells. Values represent fold-enrichment over IgG. **G** RT-qPCR analysis of *FOXA1* and *PSD4* mRNA levels in CS16 *SNAI1*-KO cells upon *FOXA1* knockdown with two independent siRNAs. Values represent fold-change of mRNA expression normalized to *GAPDH* and expressed relative to the level in siCtrl cells. **H** Representative immunoblot of three biological replicates along with molecular mass markers in kDa showing protein expression levels of ARF6 in CS16 *SNAI1*-KO cells upon FOXA1 knockdown using two independent siRNAs. GAPDH serves as loading control. Protein level quantification is shown in the lower part of the panel. *p*-values **p ≤ 0.01. ChIP-qPCR analysis for H3K27me^3^ (**I**) and H3K4me^3^ (**J**) enrichment at the *PSD4* transcription starting site in MDA-MB-231 CS16 cells upon *FOXA1* knockdown. Values in **I** and **J** represent fold-enrichment over IgG. Data in **D**, **F**, **I** and **J** are shown based on two-way ANOVA, followed by multiple paired comparisons conducted by means of Tukey’s post-test method. Data in E are presented as mean values of three biological replicates ± SEM, each in technical triplicates and *p*-values are based on a two-tailed unpaired student’s *t*-test. Data in **G** and **H** are presented as mean values of at least two biological replicates ± SEM, G in technical triplicates, and *p*-values are based on one-way ANOVA, followed by multiple paired comparisons conducted by means of Tukey’s post-test method. *p*-values: *p ≤ 0.05; **p ≤ 0.01; ****p ≤ 0.0001. All uncropped (original) immunoblots are shown as a supplemental file.

In our previous study, we found a significant upregulation of FOXA1 in *SNAI1*-KO cells that correlated with the observed intermediate epithelio-mesenchymal state and the luminal fate acquired by the cells upon loss of SNAI1 [5]. This, in combination with reports that demonstrate FOXA1 repressing TGF-β signaling in various types of cancer [39, 40], prompted us to investigate the possibility that FOXA1 is involved in the observed regulation of the PSD4 and ARF6 module. After confirming the reciprocal protein expression pattern of FOXA1 (high), and PSD4 and ARF6 (low) in the *SNAI1*-KO cells (Fig. 2A–C), and also that FOXA1 expression was not altered by TGF-β stimulation in *SNAI1*-KO cells, as previously reported [5], we rescued SNAI1 expression in the same KO cells after transient expression of the missing gene (Supplementary Fig. 1C) and compared this to SNAI1 overexpression in the parental (WT) MDA-MB-231 cells (Fig. 2D). As predicted, SNAI1 overexpression led to suppression of *FOXA1* and concomitant enhancement of *PSD4* and *ARF6* mRNA expression, not only in the KO cells but also in parental and in two additional epithelial BRCA cells, ZR-57-1 and MCF7, which express undetectable endogenous SNAI1 levels (Fig. 2D, E, and Supplementary Fig. 1D). The impact of SNAI1 overexpression on the three genes examined was proportional to the degree of SNAI1 expression (note lack of significance in *ARF6* expression in MCF7 cells), which also reflects the efficiency of transfection in each cell population. SNAI1 rescue in long-term, stably transfected cell clones led to new plasticity deviations of the overall TNBC cell phenotype and is therefore not included in this report.

ChIP-qPCR analysis in *SNAI1*-KO cells, showed significant enrichment of FOXA1 binding over the IgG control upstream from the transcriptional starting sites of both *PSD4* and *ARF6* (Fig. 2F). These data were confirmed by the enhanced FOXA1 binding to both *PSD4* and *ARF6* proximal promoter sites upon FOXA1 overexpression (induced by doxycycline) (Supplementary Fig. 1F, G), after analyzing FOXA1 ChIP-sequencing data from MCF7 cells [41]. Confirming the FOXA1 chromatin binding data functionally, transient silencing of *FOXA1* with two individual siRNAs led to derepression of *PSD4* and ARF6 in *SNAI1*-KO cells (Fig. 2G, H). Importantly, using ChIP-qPCR analysis in MDA-MB-231 *SNAI1*-KO cells, we observed a significant reduction of the promoter-associated inactive histone mark H3K27me^3^ over the *PSD4* transcription starting site upon FOXA1 knockdown, which was accompanied by a significant induction of the promoter-associated active H3K4me^3^ mark (Fig. 2I, J). Altogether, these data demonstrate that loss of SNAI1 enables FOXA1 to directly bind the *PSD4* and *ARF6* promoters and repress their transcription.

### Differential regulation of *PSD4* and *ARF6* expression by FOXA1 and TGF-β signaling

We have reported that *SNAI1*-KO cells exhibit defective TGF-β signaling [5]. We previously established that the TGF-β type II receptor is severely downregulated, yet the type I receptor is expressed at variable levels in the total cellular pool, demonstrating defective TGF-β signaling in the *SNAI1*-KO cells. We therefore performed cell surface biotinylation followed by streptavidin pulldown and monitoring of the levels of the TGF-β type I receptor (also called ALK5) (Supplementary Fig. 2A). It was readily recognized that the cell surface levels of ALK5 were significantly reduced, whereas the total receptor levels were unaltered, possibly reflecting the defects that we report on endocytic trafficking that includes receptor recycling (Fig. 1).

**Fig. 3:**
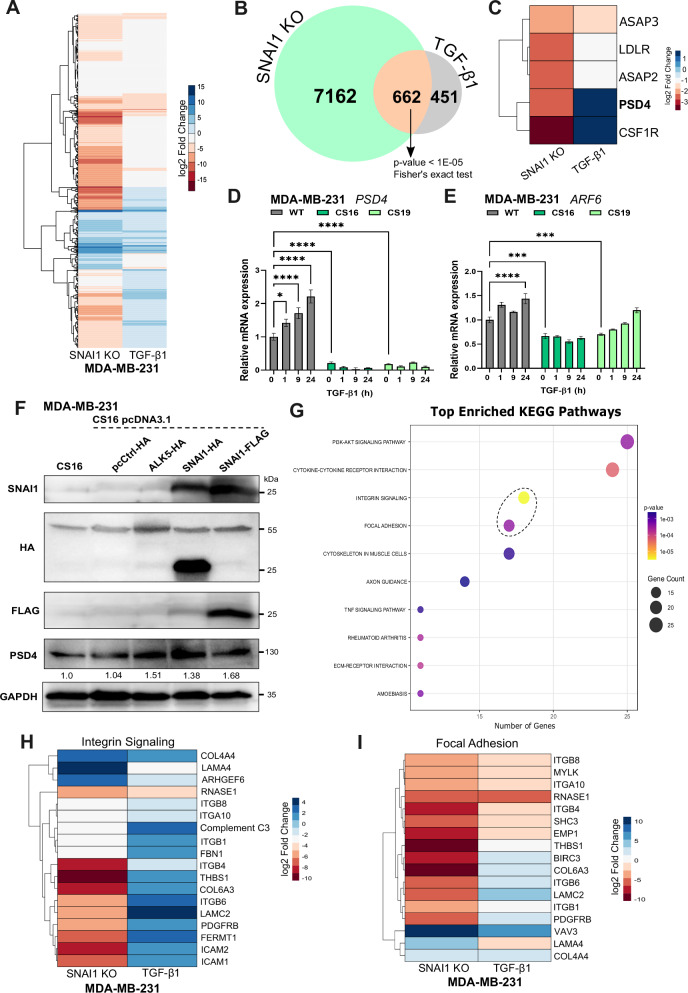
*SNAI1*-KO cells lose competence in responding to TGF-β signaling. **A** Heat map of the common 662 differentially expressed genes in the comparison of *SNAI1*-KO cells with wild-type cells stimulated with TGF-β1 for 48 h. **B** Venn diagram of the common and unique differentially expressed genes in the comparison of *SNAI1* knockout cells with wild-type cells stimulated with TGF-β1 for 48 h. **C** Heatmap demonstrating differential gene expression of the common, significantly regulated genes involved in the endocytosis molecular pathway in *SNAI1*-KO and TGF-β1 stimulated MDA-MB-231 WT cells. The color code represents normalized counts per million values of *SNAI1*-KO and TGF-β1 stimulated MDA-MB-231 WT cells; *PSD4* is highlighted. RT-qPCR analysis of *PSD4* (**D**) and *ARF6* (**E**) mRNA levels in MDA-MB-231 WT and *SNAI1*-KO cells stimulated with 5 ng/ml of TGF-β1 for the indicated time periods. Values represent fold-change of mRNA expression normalized to *GAPDH*. Data in **D** and **E** are presented as mean values of three biological replicates ± SEM, each in technical triplicates and *p*-values are based on two-way ANOVA, followed by multiple paired comparisons conducted by means of Tukey’s post-test method. *p*-values: *p ≤ 0.05; ***p ≤ 0.001; ****p ≤ 0.0001. **F** Representative immunoblot of at least two biological replicates along with molecular mass markers in kDa showing protein expression levels of endogenous SNAI1, transfected HA-tagged or FLAG-tagged SNAI1, transfected HA-tagged TGF-β type I receptor ALK5 and endogenous PSD4 proteins in CS16 *SNAI1*-KO cells upon transient transfection with empty vector (pcCtrl-HA) or the indicated three vectors. GAPDH serves as loading control and densitometry is shown for the endogenous PSD4 level responses. **G** Bubble plot of the top-enriched KEGG pathways among the differentially expressed genes that are common between *SNAI1*-KO and TGF-β1 stimulated MDA-MB-231 WT cells (shown in **A**). The color code represents *p*-values and the bubble size describes the number of genes included in each gene ontology group. A dotted oval highlights integrin and adhesion pathways. Heatmaps demonstrating differential gene expression of the common, significantly regulated genes involved in the integrin signaling (**H**) and focal adhesion (**I**) molecular pathways in *SNAI1*-KO and TGF-β1-stimulated MDA-MB-231 WT cells. These two pathways are also indicated in the bubble plot of **G**. The color code represents normalized counts per million values of *SNAI1*-KO and TGF-β1-stimulated MDA-MB-231 WT cells. All uncropped (original) immunoblots are shown as a supplemental file.

To examine deeper the involvement of TGF-β signaling in the *SNAI1*-KO phenotype, we performed RNA sequencing analysis in MDA-MB-231 WT cells stimulated with TGF-β for 48 h, recording upregulation of 528 transcripts and downregulation of 585 transcripts (log2 fold-change ±1 and FDR < 0.05) and compared their transcriptomic profiles with our previously published *SNAI1*-KO (CS16 and CS19) RNA-seq data [5]. Among the 528 upregulated transcripts, TGF-β stimulation induced *SNAI1* expression (log2 FC = 1.7 and FDR = 0.02). *SNAI1*-KO and TGF-β-stimulated cells harbored 662 commonly dysregulated transcripts showing various expression patterns (Fig. 3A, B). GSEA reproducibly showed enrichment of known signatures associated with TGF-β signaling, including EMT and TNFα signaling (Supplementary Fig. 2B). By testing the differential expression of genes (after *SNAI1*-KO or TGF-β stimulation for 48 h) involved in endocytosis based on the KEGG database, we found *PSD4* among the most significantly downregulated genes in *SNAI1*-KO cells (Fig. 3C), but not *ARF6*. Furthermore, *PSD4* expression was significantly upregulated upon TGF-β1 stimulation in WT cells and across several time points, but not in *SNAI1*-KO cells; on the other hand, *ARF6* mRNA expression showed significant variation in its expression in response to TGF-β only after 24 h, yet even *ARF6* expression was reduced upon *SNAI1* knockout (Fig. 3D, E). *ARF6* mRNA expression was induced by TGF-β in a cell type-dependent and time-dependent manner as we have recently reported [42], a pattern of response that requires further detailed analysis. TCGA dataset analysis of the TNBC-basal subtype of BRCA revealed a moderate, yet significant, positive correlation between *TGFB1* mRNA levels and *PSD4*, but not with *ARF6* (Supplementary Fig. 2C, D). Nevertheless, lower expression levels of either *PSD4* or *ARF6* mRNA predicted longer overall survival in TNBC-basal BRCA patients (Supplementary Fig. 2E), an observation that is in line with the less aggressive phenotype of the *SNAI1*-KO cells in vitro. Furthermore, re-annotation of the genomic locations of the SMAD2/3 peaks provided by our previously published ChIP-seq data, performed in human breast MCF10A-MII cells [43], showed a significant enrichment of SMAD2/3 binding to the *PSD4* promoter upon TGF-β stimulation (Supplementary Fig. 2F), a result reproduced by ChIP-qPCR analysis in MDA-MB-231 WT cells (Supplementary Fig. 2G). Moreover, the H3K4me^3^ mark that demarcates transcriptionally active promoters was significantly enriched at the *PSD4* transcription starting site in MDA-MB-231 WT cells compared to *SNAI1*-KO cells; however, TGF-β stimulation did not affect the enrichment levels (Supplementary Fig. 2H). We interpret these results as indicating that TGF-β signaling enforces SMAD2/3 binding to the *PSD4* promoter without altering the active chromatin mark at the same promoter site. In contrast, ablation of *SNAI1* drastically affects the chromatin mark, indicating profound epigenetic alteration at the *PSD4* promoter. We confirmed the effect of TGF-β signaling on PSD4 protein expression by transiently transfecting a constitutively active ALK5 receptor or two different vectors expressing SNAI1 (with an HA or a FLAG epitope) in *SNAI1*-KO cells (Fig. 3F). Reconstitution of either activated ALK5 or SNAI1 in the KO cells had a reproducible, mild effect on PSD4 protein expression. Thus, SNAI1 reconstitution led to a 1.38- or 1.68-fold upregulation of PSD4 protein (Fig. 3F), which is in the same direction as the slightly higher induction of *PSD4* mRNA measured in the *SNAI1*-KO cells under similar experimental conditions (Fig. 2D). Overall, these data demonstrate that TGF-β signaling positively regulates PSD4 but not ARF6 expression, a mechanism dominantly overtaken by the repressive activity of FOXA1 in cells that suffered the absence of SNAI1.

### Dysregulated ECM adhesive and invasive properties caused by *SNAI1* knockout

**Fig. 4:**
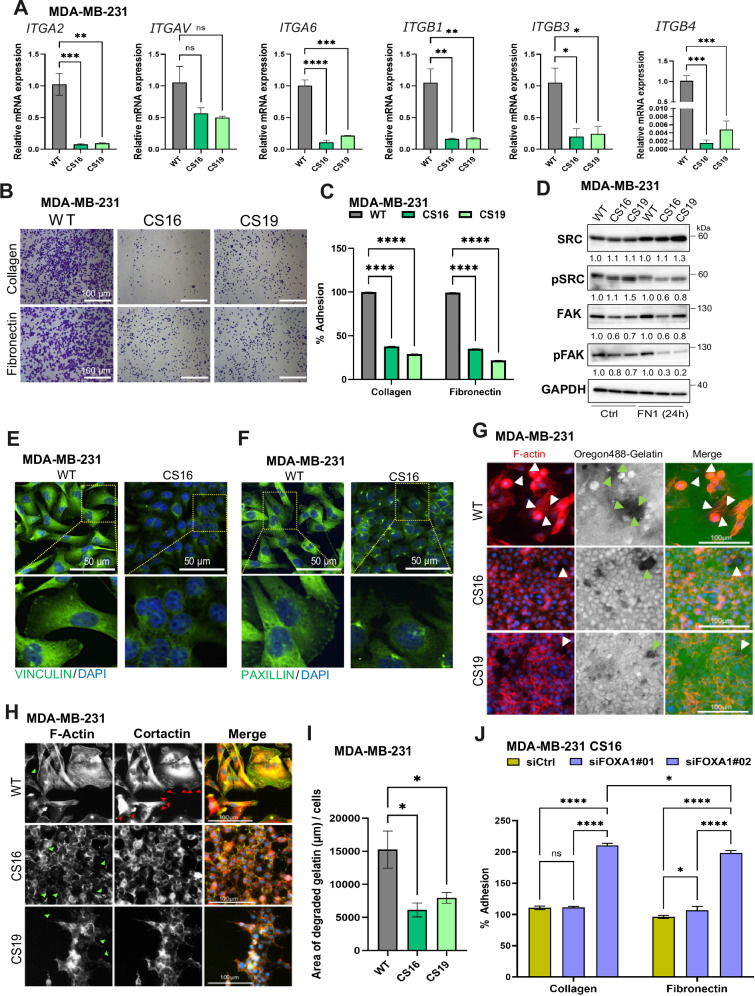
*SNAI1* knockout suppresses cell matrix adhesion and invasion. **A** RT-qPCR analysis of *ITGA2*, *ITGAV*, *ITGA6*, *ITGB1*, *ITGB3* and *ITGB4* mRNA levels in MDA-MB-231 WT and *SNAI1*-KO cells. Values represent fold-change of mRNA expression normalized to *GAPDH* and expressed relative to the level in MDA-MB-231 WT cells. Extracellular matrix-mediated cell adhesion assay in MDA-MB-231 WT and *SNAI1*-KO cells with representative images (**B**; scale bars, 100 μm) and quantification (**C**). **D** Representative immunoblot of at least two biological replicates along with molecular mass markers in kDa showing protein expression levels of SRC, pSRC, FAK and pFAK in MDA-MB-231 WT and *SNAI1*-KO cells and average densitometric analysis after adhesion to fibronectin (FN1) for 24 h. GAPDH serves as loading control. **E**, **F** Representative immunofluorescence staining images of vinculin and paxillin (green) in MDA-MB-231 WT and *SNAI1*-KO cells. Nuclei are stained in blue with DAPI. Scale bars, 50 μm. **G** Representative fluorescence images of MDA-MB-231 WT and *SNAI1*-KO cells placed on Oregon488-gelatin (green)-coated plates and stained for F-actin (red). Areas devoid of fluorescent signal indicate degradation of the fluorescent gelatin and are marked with arrowheads (with their color adjusted for clarity among images). Nuclei are stained in blue with DAPI. Scale bars, 100 μm. **H** Representative immunofluorescence staining images of cortactin (red), F-actin (green) in MDA-MB-231 WT and *SNAI1*-KO cells grown for two days in collagen type I-coated plates. Nuclei are stained in blue with DAPI. Green arrowheads indicate F-actin and red arrowheads indicate cortactin-positive regions proximal to the plasma membrane. Scale bars, 100 μm. **I** Quantification of gelatin degradation (related to **G**) calculated as the mean degradation area per total cell number ± SEM of five images. **J** Quantification of extracellular matrix-mediated cell adhesion assay in MDA-MB-231 *SNAI1*-KO cells upon FOXA1 knockdown. Data in **A** and **I** are presented as mean values of three biological replicates ± SEM, each in technical triplicates and *p*-values are based on one-way ANOVA, followed by multiple paired comparisons conducted by means of Tukey’s post-test method. Data in **C** and **J** are presented as mean values of three biological replicates ± SEM, each in technical triplicates and *p*-values are based on two-way ANOVA, followed by multiple paired comparisons conducted by means of Tukey’s post-test method. *p*-values: *p ≤ 0.05; **p ≤ 0.01; ***p ≤ 0.001; ****p ≤ 0.0001; ns not significant. All uncropped (original) immunoblots are shown as a supplemental file.

The transcriptomic data showed negative regulation of cell-matrix adhesion in *SNAI1*-KO cells (Fig. 1D). Similarly, querying the comparative transcriptomic analysis of *SNAI1*-KO cells and WT cells stimulated with TGF-β, we identified multiple GO terms concentrating around adhesion and integrin function (Fig. 3G–I). This is because TGF-β stimulation, as previously reported for different cell types, can induce expression of various integrin family members with cell type-specific and temporal patterns. Accordingly, RT-qPCR analysis confirmed the strong downregulation of six integrin family members in the *SNAI1*-KO cells (Fig. 4A), whereas transient SNAI1 overexpression in different cell models verified specific integrin receptor upregulation in a cell type-specific manner (Supplementary Fig. 3A, B). Moreover, stimulation of MDA-MB-231 WT cells with TGF-β induced the expression levels of certain integrin genes (Supplementary Fig. 3C).

To functionally validate the loss of integrin expression, we performed a cell adhesion assay in which cells were allowed to attach to fibronectin or collagen type I for 60 min and the adherent cells were quantified after staining with crystal violet. We observed a significant reduction in the adhesion of *SNAI1*-KO cells in both extracellular matrices (Fig. 4B, C), which was accompanied by reduced phosphorylation of SRC and FAK, as determined by immunoblotting using phospho-specific antibodies, especially when cells were grown on a fibronectin matrix (Fig. 4D). Repeating the adhesion assay after *SNAI1* transient overexpression in ZR-57-1 and MCF7 cells led to the reciprocal phenotype of enhanced adhesion to collagen type I or fibronectin (Supplementary Fig. 3D, E). Finally, to confirm that integrin β1 mediates adhesion of MDA-MB-231 WT cells (Supplementary Fig. 3F) to either collagen type I or fibronectin, we used the mouse monoclonal antibody P5D2 to block cell surface integrin β1 activity (Supplementary Fig. 3G). Although this inhibition reduced cell adhesion, it did not reach the markedly impaired levels observed in *SNAI1*-KO cells (Fig. 4C), underscoring the broader integrin‑less phenotype caused by the absence of SNAI1.

We next evaluated vinculin (*VCL*), a core structural component of focal adhesions, and paxillin (*PXN*), an adapter protein required for integrin-mediated FAK activation, using immunofluorescence microscopy (Fig. 4E, F). Vinculin showed a distinct expression pattern in MDA-MB-231 WT cells, characteristic of the focal adhesions formed between cells and the ECM, while vinculin was completely absent from *SNAI1*-KO cells. Paxillin showed a similar pattern as vinculin in MDA-MB-231 WT cells, while in *SNAI1*-KO cells it seemed to be localized around the periphery of the nuclear envelope. Accordingly, the RNA-seq analysis showed that *VCL* was significantly downregulated (log2 FC = −1.62 and FDR = 4.5E-19), whereas *PXN* was downregulated but not significantly (log2 FC = −3.9 and FDR = 0.2). Interestingly, unlike WT cells, *SNAI1*-KO cells showed no increase in FAK phosphorylation after stimulation with TGF-β, attesting to the defective TGF-β signaling in the absence of SNAI1 (Supplementary Fig. 3H; note that these experiments were performed in the presence of low serum, revealing a more dramatic difference in FAK protein levels between WT and *SNAI*-KO cells, because the stabilizing effect of serum on FAK is removed).

Since adhesive processes and the functions of PSD4 and ARF6 are involved in cell invasion through the basement membrane (see Introduction), we next explored whether *SNAI1*-KO cells exhibit altered matrix degradative properties. Using fluorescent gelatin degradation assays, we observed that *SNAI1*-KO cells exhibited significantly reduced ability to degrade the ECM compared to their WT counterparts, visible as fewer and smaller dark degradation zones beneath the cells (Fig. 4G, arrowheads; 4I, quantification). Consistent with these data, when *SNAI1*-KO cells adhered to collagen type I, they presented an abnormal cortactin distribution (Fig. 4H). Cortactin, a well-known marker for invadopodia, was recruited to the cell cortex of WT cells, where it co-stained with F-actin (Fig. 4H, red arrowheads). On the contrary, cortactin in *SNAI1*-KO cells exhibited mostly a cytoplasmic expression pattern with a less pronounced cortical localization and co-staining with cortical actin filaments (Fig. 4H, merge). Unexpectedly, in the same *SNAI1*-KO cells when cultured on collagen type I, F-actin staining demonstrated frequent formation of pronounced filopodia in a pattern indicative of cell movement (Fig. 4H, green arrowheads). WT cells rarely exhibited such extended filopodial formations (Fig. 4H, WT, green arrowhead).

Searching for metalloproteinases responsible for matrix degradation and invasion, the transcriptomic data identified *MMP14*/MT1-MMP among the most downregulated MMPs (Supplementary Fig. 4A). Interestingly, MMP14 has been described as the main metalloproteinase involved in mammary gland morphogenesis and BRCA metastasis [44, 45] and its downregulated levels were further validated in the *SNAI1*-KO cells (Supplementary Fig. 4B). These results demonstrate that loss of SNAI1 leads to reduced cell-matrix adhesion accompanied by fewer degradative invadopodia that lack expression of *MMP14*.

Inducible FOXA1 ChIP-sequencing data from MCF7 cells [41] showed that FOXA1 binds the *MMP14* gene promoter, as well as the *ITGA1*, *ITGA2* and *ITGB3* genomic regions (Supplementary Fig. 4C). Guided by these observations, we tested the effects of *FOXA1* knockdown and found that it significantly enhanced *MMP14* mRNA expression, whereas not all integrin genes responded to the *FOXA1* silencing in a coordinate manner (Supplementary Fig. 4D). We interpret these results with caution because the ChIP-sequencing data are derived from MCF7 cells and our gene expression analysis is derived from MDA-MB-231 cells. This implies that FOXA1, in a cell type-dependent manner, can regulate distinct genes of the integrin family, a hypothesis worth investigating across the integrin family and in multiple breast cancer cell models. Despite evident variations in the regulation of specific adhesion genes, *FOXA1* depletion also increased SRC and FAK phosphorylation (Supplementary Fig. 4E), key actin-regulating events downstream of integrin receptors, and adhesion to collagen or fibronectin (Fig. 4J, note that FOXA1 siRNA#02, whose impact on ARF6 expression is stronger (Supplementary Fig. 4D), generates a significant impact on cell adhesion to fibronectin but not to collagen type I). Altogether, these findings potentially indicate that FOXA1 suppresses integrin-based signaling and expression of specific metalloproteinases in TNBC-basal cells. The impact of SNAI1 and FOXA1 on cell adhesion most likely implicates integrin signaling and the functions of PSD4 and/or ARF6, yet direct transcriptional regulation of integrin family genes by SNAI1/FOXA1 may not be a critical factor in this process.

### *SNAI1* knockout reduces endosomal/lysosomal vesicular maturation

**Fig. 5:**
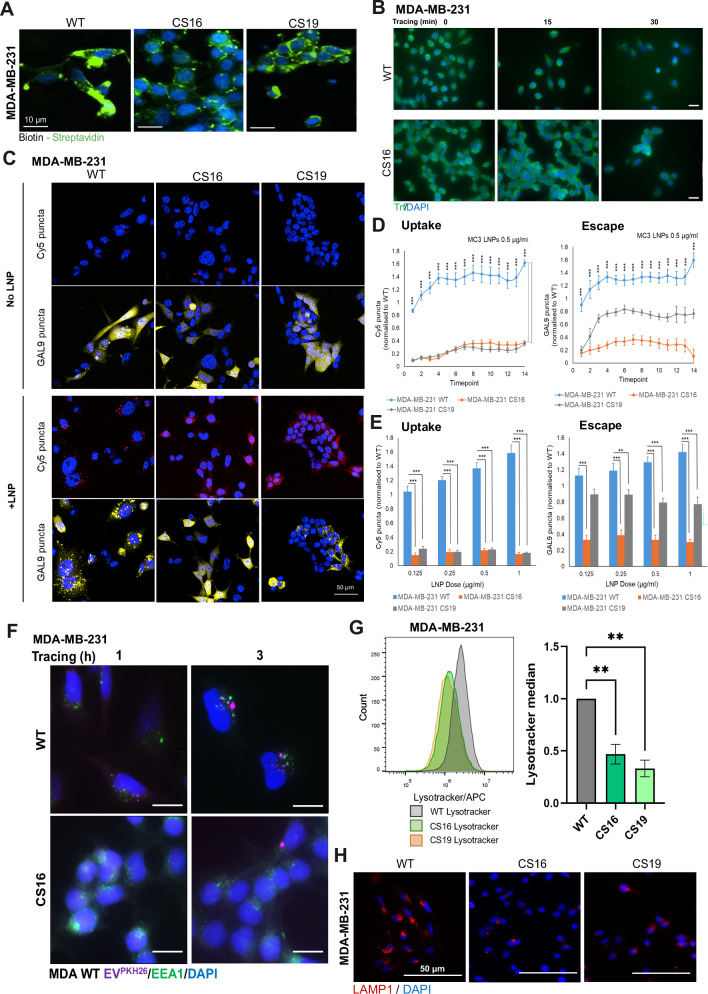
Loss of SNAI1 suppresses receptor-mediated endocytosis and vesicular uptake. **A** Representative fluorescence images taken at 60 min after initiating endocytosis of labeled biotin in MDA-MB-231 WT and *SNAI1*-KO cells. Cells were treated with FITC-labelled streptavidin to detect endocytosed biotinylated proteins. Nuclei are stained in blue with DAPI. Scale bars, 10 μm. **B** Transferrin (Trf) uptake fluorescence intensity in MDA-MB-231 WT and *SNAI1*-KO cells obtained after tracing for the indicated time points. Scale bars, 25 μm. **C** Representative images of stably mCherry-GAL9 expressing MDA-MB-231 WT and *SNAI1*-KO cells imaged by live-cell microscopy, 5 h post-dosing with 0.5 μg/ml MC3-LNPs. Scale bars, 50 μm. **D** Kinetic analysis of Cy5-mRNA and mCherry-GAL9 puncta formed across 0–14 h of incubation with 0.5 μg/ml MC3-LNPs in mCherry-GAL9 MDA-MB-231 WT and *SNAI1*-KO cells. Values indicate LNP uptake (Cy5-mRNA puncta) and endosomal escape (mCherry-GAL9 puncta). Data are presented as mean values of three biological replicates ±SEM and *p*-values for each time point are shown based on two-tailed unpaired student’s *t*-test. **E** Comparison of LNP uptake (Cy5-mRNA puncta) and endosomal escape (mCherry-GAL9 puncta) in mCherry-GAL9 MDA-MB-231 WT and *SNAI1*-KO cells 5 h after incubation with MC3 LNPs across 0.125–1.0 μg/ml doses. Data are presented as mean values of three biological replicates ±SEM and *p*-values (**p ≤ 0.01, ***p ≤ 0.001) are shown based on two-tailed unpaired student’s *t*-test. **F** Uptake of vital-labeled EVs with PKH26 along with immunofluorescence microscopy of endogenous EEA1 and nuclei (DAPI) in MDA-MB-231 WT and *SNAI1*-KO cells obtained after tracing at the indicated time points. Scale bars, 20 μm. **G** Overlay of representative lysotracker fluorescence intensity histogram in MDA-MB-231 WT and *SNAI1*-KO cells, as obtained by flow cytometry analysis and corresponding quantification of the flow cytometry data. Data are presented as mean values of three biological replicates ± SEM and *p*-values (**p ≤ 0.01) are shown based on one-way ANOVA, followed by multiple paired comparisons conducted by means of Tukey’s post-test method. **H** Representative immunofluorescence images of LAMP1 (red) in MDA-MB-231 WT and *SNAI1*-KO cells. Nuclei are stained in blue with DAPI. Scale bars, 50 μm.

To investigate the impact of SNAI1 loss on the endocytic pathway, the second major phenotype proposed by our RNA-seq analysis (Fig. 1), we initially performed a surface biotinylation assay. In this assay, cells were incubated on ice with membrane-impermeable sulfo-NHS-SS-biotin and subsequently transferred to 37 °C to initiate endocytosis. Fluorescent streptavidin staining of the biotinylated proteins showed that *SNAI1* knockout reduced the intracellular transport, with most biotinylated proteins remaining at the cell periphery (Fig. 5A). To further establish this defect, we explored transferrin receptor (TfR) recycling, whose expression is not affected by the absence of SNAI1 (Fig. 5B and Supplementary Fig. 5A). MDA-MB-231 cells were incubated with Alexa488-conjugated Trf on ice for 45 min, washed to remove unbound ligand, and then shifted to 37 °C for 15 min to allow internalization (0 min). Cells were subsequently returned to ice and treated with a quenching agent to eliminate residual surface‑bound fluorescence, followed by incubation in complete medium containing holo‑Trf to visualize retained, chased TfR. After 30 min, most labeled TfR had been recycled in WT, but not in *SNAI1*-KO cells, as indicated by reduced cytoplasmic fluorescence (Fig. 5B).

As a complementary and clinically relevant means to monitor clathrin-mediated endocytosis, we utilized lipid nanoparticles (LNPs), since LNPs absorb serum components that act as surface ligands that bind to recipient cell receptors, especially low-density lipoprotein receptors [46]. The LNPs were loaded with Cy5-mRNA, enabling monitoring of cellular internalization and trafficking. LNP-cargoes traffic from early to late endosomes, then are delivered to the cytoplasm via effectively remodeled endosomal (escaping) vesicles, a process marked by the association of members of the galectin family of proteins with these vesicles, or the cargoes are ultimately degraded in lysosomes [46, 47]. Upon remodeling of the endosomal membrane that is induced by ionizable lipids formulated within the LNPs, here Dlin-MC3-DMA (MC3), galectins that are primarily expressed in the cytoplasm are recruited to endosomes where they bind to exposed β-galactosides, a process naturally serving cell defense against internalized pathogens [48–51]. Here, we utilized galectin-9 (GAL9) recruitment as a readout of endosomal escape [38]. Stable MDA-MB-231 WT and *SNAI1*-KO mCherry-GAL9 reporter cells were treated with MC3 LNPs carrying Cy5-labeled mRNA to assess cellular uptake, as well as endosomal escape. Punctate Cy5 signals detected after 5 h of incubation indicated MC3-LNP uptake, whereas initially diffused cytoplasmic mCherry-GAL9 fluorescent signals formed punctate structures, indicative of endocytic escape in WT cells (Fig. 5C). *SNAI1*-KO cells presented less Cy5-mRNA and mCherry-GAL9 puncta, as attested also after a 14 h-long quantification of the two signals (Fig. 5C–E). MC3-LNP dose-response experiments aimed at verifying that formation of mCherry-GAL9 puncta correlated with the dose of MC3-LNPs used. Whilst WT cells presented a dose-dependent increment in punctate structures 5 h after incubation with MC3-LNPs, the punctate structures remained low and unaffected by LNP dose in the *SNAI1*-KO cells (Fig. 5D, E).

Using an alternative internalization assay, we assessed the uptake of EVs secreted by MDA-MB-231 WT cells, enriched as the VSF and labeled with the lipophilic compound PKH26 (Supplementary Fig. 5B). This assay revealed impaired EV uptake in *SNAI1*-KO cells compared to WT cells (Fig. 5F), which could be attributed to defective endocytic machinery. Furthermore, we investigated the maturation of endosomes to lysosomes and found that *SNAI1*-KO cells contained significantly less lysosomes, as indicated by the decreased lysotracker signals, and decreased LAMP1 protein levels (Fig. 5G, H, and Supplementary Fig. 5C). Thus, *SNAI1*-KO cells present a general defect in receptor-mediated internalization of diverse natural and synthetic proteins or extracellular nanoparticles, emphasizing the validity of the original transcriptomic gene-ontology profile.

### *SNAI1* knockout reduces EV release

**Fig. 6:**
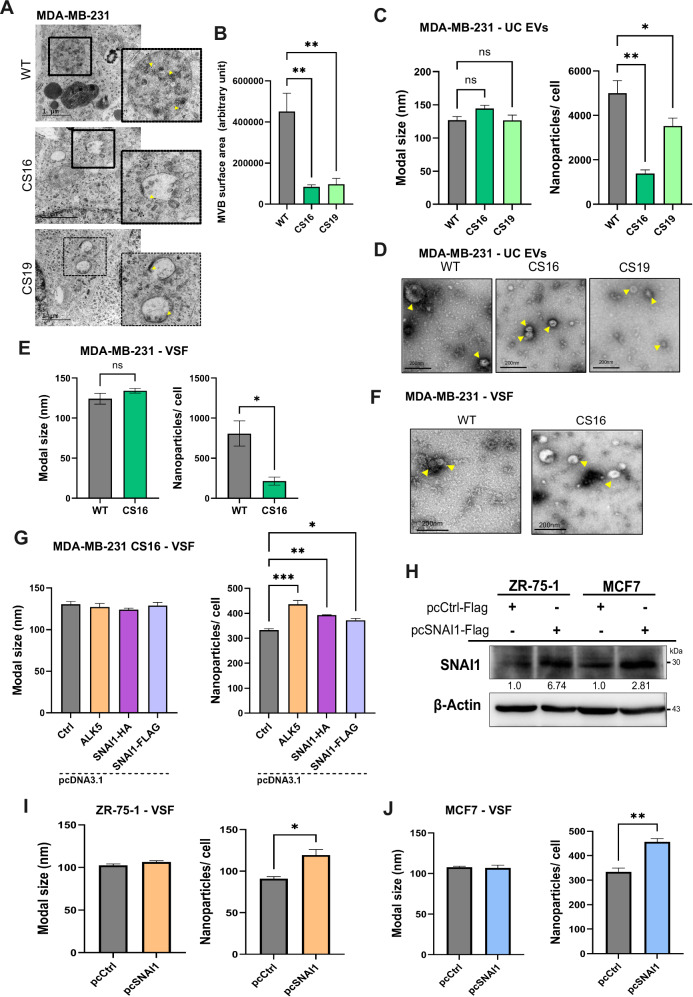
Defective multivesicular body architecture and extracellular vesicle release upon SNAI1 knockout. **A** Representative TEM pictures taken at 3 h after initiating endocytosis of gold-labeled BSA in MDA-MB-231 WT and *SNAI1*-KO cells. Scale bars, 1 μm. **B** Quantification of the cross-section area of the MVBs as calculated by Image J analysis. Nanoparticles released by MDA-MB-231 WT and *SNAI1*-KO cells collected after ultracentrifugation (UC EVs, (**C**)) or vesicular secretory fraction (VSF, (**E**)) as quantified by NTA, reporting the size of the nanoparticles with the highest frequency (mode) within the sample and their numbers normalized to the total cell number. Representative TEM pictures depicting the morphology and the size of MDA-MB-231 WT and *SNAI1*-KO derived particles (UC EVs (**D**) and VSF EVs (**F**)). Scale bars, 200 nm. **G** Nanoparticles released by MDA-MB-231 *SNAI1*-KO cells transiently transfected with empty vector (Ctrl), vector expressing the TGF-β type I receptor ALK5 or two epitope-tagged (HA- or FLAG-) forms of SNAI1 (rescue experiment), collected as VSF and quantified by NTA reporting the size of the particles with the highest frequency (mode) within the sample and their numbers normalized to the total cell number. **H** Representative immunoblot and average densitometric analysis of at least two independent biological replicates along with molecular mass markers in kDa showing protein expression levels of SNAI1 in ZR-57-1 and MCF7 cells upon FLAG-SNAI1 overexpression. β-Actin serves as a loading control. Nanoparticles released by ZR-57-1 (**I**) or MCF7 (**J**) cells transiently transfected with empty vector (Ctrl) or vector expressing an epitope-tagged (FLAG-) form of SNAI1 (pcSNAI1, rescue experiment), collected as VSF and quantified by NTA reporting the size of the particles with the highest frequency (mode) within the sample and their numbers normalized to the total cell number. Data in **B**, **C** and **G** are presented as mean values of at least two biological replicates ± SEM, each in technical duplicates and *p*-values are based on one-way ANOVA, followed by multiple paired comparisons conducted by means of Tukey’s post-test method. Data in **E**, **I** and **J** are presented as mean values of at least two biological replicates ± SEM, each in technical duplicates and *p*-values are based on unpaired *t*-test with Welch’s correction. *p*-values: *p ≤ 0.05; **p ≤ 0.01; ***p ≤ 0.001; ns not significant. All uncropped (original) immunoblots are shown as a supplemental file.

Another key process associated with endocytic trafficking is the secretion of exosomes, which are generated through vesicular sorting within MVBs [29]. Accordingly, late endosomes can either fuse with lysosomes for cargo degradation or traffic their intraluminal vesicles to the plasma membrane for release into the extracellular space. To this end, we identified MVBs of MDA-MB-231 cells by TEM after endocytosis of gold-BSA and demonstrated that *SNAI1*-KO cells contained smaller MVBs with less intraluminal vesicles when compared to their WT counterparts (Fig. 6A, B). Moreover, a significantly lower number of EVs was released from *SNAI1*-KO cells (estimated by two complementary EV enrichment methods, ultracentrifugation - UC and VSF), further validating a decrease in MVB-derived vesicles, while the EV modal size was not affected (Fig. 6C–F). Morphological evaluation of EVs by TEM confirmed the presence of membrane vesicles of 50–200 nm in diameter, but no obvious differences in EV morphology (Fig. 6D, F). Rescuing *SNAI1* expression in the *SNAI1*-KO clone CS16 by two independent SNAI1 vectors or rescuing defective TGF-β signaling by transfecting the constitutively active ALK5 receptor, led to a small, yet significant increase in EV secretion (Fig. 6G, and Supplementary Fig. 6A, B). Similarly, overexpression of SNAI1 in ZR-75-1 and MCF7 cells that express very weak endogenous SNAI1 levels led to enhanced release of EVs (Fig. 6H–J, and Supplementary Fig. 6C). These results suggest that SNAI1 controls molecular networks that regulate EV release.

### EV release is negatively regulated by FOXA1 and positively regulated by ARF6

**Fig. 7:**
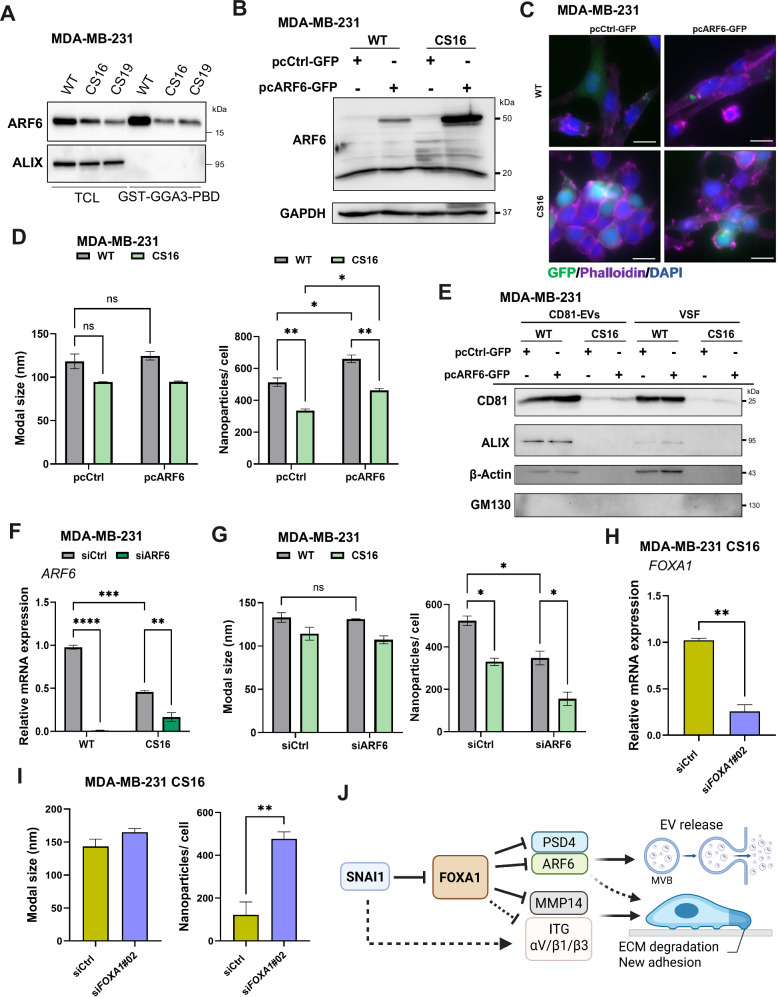
FOXA1 and ARF6 regulate EV secretion. **A** Representative immunoblot of at least two biological replicates along with molecular mass markers in kDa showing protein expression levels in total cell lysates (TCL) and after pulldown using GST-GGA3-PBD domain protein, capturing the GTP-bound, active ARF6 form in MDA-MB-231 WT and *SNAI1*-KO cells. ALIX serves as a loading control in the TCL. **B** Representative immunoblot of at least two biological replicates along with molecular mass markers in kDa showing protein expression levels of GFP-tagged ARF6 (~50 kDa) after transient transfection of MDA-MB-231 WT and *SNAI1*-KO cells with GFP-alone vector (pcCtrl-GFP) and pcARF6-GFP. GAPDH serves as a loading control. **C** Representative immunofluorescence images of ARF6-GFP (green) and direct fluorescence of F-actin (magenta, phalloidin) in MDA-MB-231 WT and *SNAI1*-KO cells. Nuclei are stained in blue with DAPI. Scale bars, 50 μm. **D** Nanoparticles released by MDA-MB-231 WT or CS16 *SNAI1*-KO cells upon transient expression of GFP vector (pcCtrl) and ARF6-GFP (pcARF6) as quantified by NTA, reporting the size of the particles with the highest frequency (mode) within the sample and EV numbers normalized to the total cell number. **E** Representative immunoblot of three biological replicates along with molecular mass markers in kDa showing protein expression levels of EV marker proteins (CD81, ALIX, β-Actin) and negative control (Golgi-specific GM130), in the same EV fractions (after CD81-specific affinity enrichment or in crude VSF) used in **D**, collected after transient transfection of MDA-MB-231 WT and *SNAI1*-KO cells with GFP-alone vector (pcCtrl-GFP) or pcARF6-GFP. **F** RT-qPCR analysis of *ARF6* mRNA levels in MDA-MB-231 WT and *SNAI1*-KO cells upon *ARF6* knockdown. Values represent fold-change of mRNA expression normalized to *GAPDH* and expressed relative to the level in siCtrl cells. **G** Nanoparticles released by MDA-MB-231 WT or CS16 *SNAI1*-KO cells upon *ARF6* knockdown as quantified by NTA, reporting the size of the nanoparticles with the highest frequency (mode) within the sample and EV numbers normalized to the total cell number. **H** RT-qPCR analysis of *FOXA1* mRNA levels in MDA-MB-231 *SNAI1*-KO cells upon *FOXA1* knockdown. Values represent fold-change of mRNA expression normalized to *GAPDH* and expressed relative to the level in siCtrl cells. **I** Nanoparticles released by CS16 *SNAI1*-KO cells upon *FOXA1* knockdown as quantified by NTA and reporting the size of the nanoparticles with the highest frequency (mode) within the sample and their normalized number to the total cell number. **J** Schematic illustration of the transcriptional network regulated by SNAI1 and FOXA1, leading to regulation of EV release, cell-ECM adhesion and ECM degradation that facilitates motility and invasiveness of cancer cells. Dotted (positive or negative arrows) indicate ambitious regulatory connections that are not fully elucidated in this study. Data in **D**, **F**, **G** and **I** are presented as mean values of three biological replicates ± SEM, each in technical triplicates and *p*-values are based on two-way ANOVA, followed by multiple paired comparisons conducted by means of Tukey’s post-test method. Data in **H** are presented as mean values of three biological replicates ± SEM, and *p*-value is based on unpaired *t*-test with Welch’s correction. *p*-values: **p ≤ 0.01; ***p ≤ 0.001; ****p ≤ 0.0001; ns not significant. All uncropped (original) immunoblots are shown as a supplemental file.

Among such factors, we first confirmed that ARF6 expression level and its GTP-bound (i.e., active form) were significantly reduced in the *SNAI1*-KO cells relative to their WT counterparts (Fig. 7A). Transient overexpression of a wild-type GFP-tagged ARF6 protein in both WT and *SNAI1*-KO cells (Fig. 7B), resulted in physiologically relevant localization of ARF6 in membrane- and actin cytoskeleton-proximal structures (Fig. 7C), and most importantly, the number of released EVs was significantly enhanced in both cell types (Fig. 7D), whereas the EVs secreted by *SNAI1*-KO cells transfected with ARF6-GFP was statistically indistinguishable from the numbers secreted from parental MDA-MB-231 WT cells (Fig. 7D). The EVs analyzed in these assays had the standard morphological features of EVs secreted by MDA-MB-231 cells as assessed by TEM (Supplementary Fig. 6D). The presence of classical EV-positive (CD81, ALIX and β-Actin) protein markers and the absence of the EV-negative (GM130) marker, analyzed on equal sample volumes of VSF or CD81-positive EVs (CD81-EV), confirmed the reduced EV secretion in *SNAI1*-KO cells compared to MDA-MB-231 WT cells (Fig. 7E). Notably, ARF6-GFP overexpression increased the abundance of CD81-EVs secreted by both WT and *SNAI1*-KO cells, consistent with the NTA data. Conversely, silencing endogenous ARF6 in WT or *SNAI1*-KO MDA-MB-231 cells significantly decreased EV release (Fig. 7F, G). Notably, since *SNAI1*-KO cells already express lower ARF6 levels and release fewer EVs, further ARF6 silencing led to an even more severe reduction in ARF6 expression and EV secretion. Finally, transient silencing of *FOXA1* (Fig. 7H, and Supplementary Fig. 4D) significantly induced EV release in *SNAI1*-KO cells (Fig. 7I, and Supplementary Fig. 6E), supporting the molecular model of its repression by *SNAI1*. Our data demonstrate that SNAI1 plays an unexpected role in modulating EV release by tumor cells, and at least one of the key components in this mechanism is FOXA1, by transcriptionally repressing *PSD4* and *ARF6*. Among these cooperating factors, ARF6 appears to be a critical effector for the latter regulation of EV secretion by SNAI1.

## Discussion

Dynamic alterations of cell-cell adhesion, including dis- and re-assembly of key adhesive complexes, cytoskeletal reorganization and remodeling of secreted extracellular proteins leading to new cell-matrix interactions characterize the EMTs [2]. It is only logical, though not well-investigated, that such cell biological processes may also involve the regulation of vesicular trafficking by EMT-TFs that could coordinate endocytic to degradative and secretory processes in the remodeled cellular phenotype. In the current study, we provide evidence that SNAI1 has a critical role in regulating integrin-based cell-matrix adhesion and invadopodial-metalloproteinase-driven cell invasion, while also regulating endocytic dynamics and EV release. Our work deciphers a key pathway that mediates the action of SNAI1, via its downstream transcription factor FOXA1, which is repressed by SNAI1. FOXA1 further impacts cell adhesion to ECM and endocytic dynamics that also couple to EV secretion (Fig. 7J). We and others have shown that SNAI1 regulates the TGF-β/SMAD signaling pathway in TNBC-basal BRCA cells and is a strong inducer of EMT and cell-matrix interactions that promote invasion [5, 37, 52]. As explained in our earlier report [5], the knockout of *SNAI1* in MDA-MB-231 cells led to a severe defect in the expression of TGF-β receptors, thus compromising the potency of these cells to respond to signals by this important cytokine in the context of the tumor microenvironment. By reproducing aspects of these results here, focusing on cell surface TGF-β type I receptor pools, and based on the loss of expression of multiple integrin receptors, we propose that the overall defect in vesicular trafficking impacts multiple pathways that involve cell surface receptors, independent of their putative function as signaling mediators, adhesion connectors, or regulators of endocytic processes.

The newly deciphered mechanistic model involves the established function of SNAI1 as a nuclear transcriptional repressor [53]. In a similar cellular context, the EMT-TF ZEB1 has been shown to induce endosomal trafficking to alter cell polarity and induce motility of lung adenocarcinoma cells [28]. The fact that MDA-MB-231 cells upon *SNAI1* knockout exhibited defects in both the uptake of artificial LNPs and of natural, homologous EVs generated by pools of the same cell type, pointed to defective mechanisms at the level of MVBs, but also at the level of late endosomal to lysosomal maturation, which correlated with reduced EV secretion by the same knockout cells. In agreement with these observations, in hepatocellular carcinoma cells, the lysosomal protein LAMP2 signals the transcriptional repression of the *SNAI1* gene [54], suggesting a reciprocal or mutual regulatory connection between SNAI1 and lysosomal function. Furthermore, impaired lysosomal function has been reported to lead to increased EV secretion as a possible way by which the cell seeks to maintain its homeostasis [55, 56]. These examples underscore the necessity for deeper investigation of this network of vesicular transport mechanisms controlled by SNAI1.

Based on the observed repression of *FOXA1* by SNAI1 in TNBC-basal BRCA cells, SNAI1 positively regulates the small GTPase ARF6 and at least one of its regulatory components, the PSD4 exchange factor. Our analysis of *SNAI1*, *FOXA1*, *PSD4* and *ARF6* expression in various carcinomas, including BRCA cells, demonstrated that the magnitude of their expression does not follow the specificity of the tissue type but most probably reflects the specific oncogenic events and epigenetic alterations that each tumor type exhibits. In relation to this finding, it is interesting that the pioneering TF FOXA1 [57], whose function is necessary for mammary ductal epithelial differentiation and organogenesis [58], represses basal BRCA phenotypes [59]. In parallel, FOXA1 is expressed in several epithelial tissues [57] and associates with cell-type-specific loci across the genome by cooperating with specific histone modifications in nucleosomes and with lineage-specific TFs [60, 61]. This suggests that genes such as *PSD4* and *ARF6* (but possibly even *MMP14* and distinct *integrins* (*ITG*)) are regulated downstream of SNAI1 and FOXA1 transcriptional signals (Fig. 7J), via distinct mechanisms that may involve different cooperating TFs. If this hypothesis is correct, the regulation of the above genes may exhibit cell type-specific patterns in different carcinomas, which is worth investigating deeper across multiple carcinoma types. The mechanism that involves PSD4/ARF6 is also corroborated by the analysis of focal adhesion proteins, such as paxillin and vinculin. This is based on the evidence that delivery of paxillin from the perinuclear space to focal adhesions depends on ARF6, a mechanism that facilitates cell motility [62]. Thus, in the absence of SNAI1, loss of cell motility appears to be coupled to the regression of paxillin into the perinuclear space, potentially due to the defective PSD4/ARF6 function. Moreover, since PSD4 and ARF6 were previously linked to EV biogenesis and secretion as well as to the regulation of invadopodial formation [23, 26, 29, 63], the SNAI1-dependent regulation of *FOXA1* and downstream *PSD4*, *ARF6* and *integrin* genes can partially explain the effects on EV secretion and invadopodial invasion. While the ARF6 gain-of-function experiments support a role of this GTPase in promoting EV secretion downstream of SNAI1 and FOXA1, equivalent gain-of-function studies for PSD4 will be necessary to establish the possible role of this enzyme in ARF6-mediated EV release. Given that PSD4 recruits ARF6 to specific plasma-membrane microdomains via its lipid‑binding pleckstrin homology domain, thereby activating ARF6 at the cell cortex, PSD4 may, in fact, spatially constrain or fine‑tune ARF6 activity [64]. Consequently, PSD4 could either promote or potentially limit ARF6‑dependent EV secretion, depending on how its localization and regulatory functions influence ARF6 signaling dynamics. This is in agreement with evidence whereby ARF6 links endocytic processes by specific adaptor proteins to the downstream function of specific phospholipases that control the secretion of EVs [65], additional mechanisms worth exploring further in the context of EMT-TFs.

The defective cell adhesion to the ECM was accompanied not only by reduced *integrin* expression levels but also by reduced SRC and FAK phosphorylation. Interestingly, inhibitors of the kinase activities of FAK or SRC have been shown to reverse EMT by inducing expression of the epithelial adherens junction protein E-cadherin and promoting cell growth in compact epithelial-like cell colonies [66, 67]. ARF6 also operates as an effector of SRC in mediating EV secretion [65]. Furthermore, in addition to the downmodulation of integrins, the equivalent loss of key components of the ECM, such as the biosynthetic enzyme HAS2 (Fig. 1D) that generates hyaluronan in breast cancer cells, may provide additional clues for a coordinated role of extracellular and intracellular signaling mediators that impact the crosstalk between adhesion and EV uptake.

In conclusion, this study sheds light on mechanisms that control endocytic vesicular trafficking networks and EV biogenesis by transcriptional pathways. It also deepens our understanding of the importance of vesicular trafficking programs that affect cell endocytic alterations and cell-matrix interactions during EMT. We propose that SNAI1 represses FOXA1 expression and drives a vesicular trafficking program that contributes to enhanced cell-matrix interactions and invasiveness, as well as poor survival of TNBC patients. Further studies of this signaling network regulated by SNAI1 (Fig. 7J), and possibly additional EMT-TFs, will advance our understanding of the progression of TNBC‑basal BRCA toward highly invasive and metastatic disease and may reveal opportunities for therapeutic intervention.

## Supplementary information

Supplemental File

Supplemental Uncropped Immunoblots

## Data Availability

The primary RNA-sequencing data of *SNAI1* knockout cells have been published [5] and deposited to GEO (accession number GSE210870). The primary RNA-sequencing data of WT MDA-MB-231 cells stimulated with TGF-β are deposited to GEO (accession number GSE329969). The additional experimental resources used and/or analyzed during the current study are available from the corresponding author on reasonable request.
