# Supplementary material for: SNAI1 ablation alters integrin-mediated adhesion and endocytic fate
Source: Cell Death Dis. 2026 Aug 12;17(1):716. doi: 10.1038/s41419-026-09179-x (PMC13470233; doi:10.1038/s41419-026-09179-x)
Supplement: Supplementary file 1 [file 41419_2026_9179_MOESM1_ESM.pdf]

SNAI1 ablation alters integrin-mediated adhesion and endocytic fate

Chrysoula Tsirigoti[1,2], Mohamad Moustafa Ali[1], Anita Morén[1], Staffan Johansson[1], Michael J. Munson[2], Carl-Henrik Heldin[1], Aristidis Moustakas[1,§] and Dorival Mendes Rodrigues-Junior[1]

SUPPLEMENTARY INFORMATION

SUPPLEMENTAL TABLES

Supplementary Table S1. List of antibodies used at the indicated dilutions, applications, vendors and catalogue numbers.

Antibody	Source	Specification
anti-ALIX<br>(1:500 immunoblotting)	Santa Cruz Biotechnology Inc.	Cat# sc-53540
anti-CD81<br>(1:500 immunoblotting)	Santa Cruz Biotechnology Inc.	Cat# sc-166029
anti-LAMP1<br>(1:500 immunoblotting)<br>(1:100 immunofluorescence)	BD Biosciences	Cat# 51-9002014
anti-SRC (36D10)<br>(1:1 000 immunoblotting)	Cell Signaling Technology	Cat# 2109
anti-SRC pY419<br>(1:1 000 immunoblotting)	Cell Signaling Technology	Cat# 2101
anti-FAK<br>(1:1 000 immunoblotting)	BD Biosciences	Cat# 610087

anti-FAK pY397<br>(1:1 000 immunoblotting)	BD Biosciences	Cat# 611722
anti-CORTACTIN (p80/85, clone 4F11)<br>(1:100 immunofluorescence)	Sigma-Aldrich AB	Cat# 05-180
anti-VINCULIN<br>(1:100 immunofluorescence)	Sigma-Aldrich AB	Cat# V9131
anti-PAXILLIN<br>(1:100 immunofluorescence)	BD Biosciences	Cat# P13520
anti-PSD4<br>(1:500 immunoblotting)	ThermoFisher Scientific	Cat# PA5-31837
anti-ARF6 (D12G6)<br>(1:1 000 immunoblotting)	Cell Signaling Technology	Cat# 5740
anti-FOXA1<br>(1:1 000 immunoblotting)	Abcam, Cambridge, UK	Cat# ab23728
anti-FOXA1<br>(1 µg/mg lysate, ChIP)	Abcam, Cambridge, UK	Cat# ab23728
anti-SMAD2/3 (Clone 18 (RUO))<br>(1 µg/mg lysate, ChIP)	BD Biosciences	Cat#610843
anti-SNAI1 (1:200 immunoblotting)	Cell Signaling Technology	Cat#3879T

anti-HA (C29F4) (1:5 000 immunoblotting)	Cell Signaling Technology	Cat# 3724
anti-FLAG (DYKDDDDK) Tag (1:5 000 immunoblotting)	Thermo Fisher Scientific	Cat#MA1-91878
anti-EEA1 (1:150, immunofluorescence)	BD Bioscences	Cat#610456
anti-ALK5 (1:1 000 immunoblotting)	Abcam	Cat#ab31013
anti-TfR (1:1 000 immunoblotting)	Cell Signaling Technology	Cat# #13113
Alexa Fluor® 488 anti-human CD29/ Integrin β1 (1:150, immunofluorescence)	Biolegend	Cat#303016
anti-ALIX/HP95 (1:2 000 immunoblotting)	Sigma-Aldrich AB	Cat# HPA011905
anti-GAPDH (6C5) (1:2 000 immunoblotting)	Thermo Fisher Scientific	Cat# AM4300
anti-β-actin (AC-15) (1:5 000 immunoblotting)	Santa Cruz Biotechnology Inc.	Cat# sc-69879

anti-Rabbit IgG H+L) Secondary, HRP	Thermo Fisher Scientific	Cat# 65-6120
anti-Mouse IgG (H+L) Secondary, HRP	Thermo Fisher Scientific	Cat# 62-6520
anti-mouse Alexa 488 Fluor	Thermo Fisher Scientific	Cat# A21202
anti-mouse Alexa 546 Fluor	Thermo Fisher Scientific	Cat# A11030

Supplementary Table S2. List of oligonucleotides (Fw, forward; Rev, reverse)

**mRNA-specific RT-PCR primers**	**Sequence**
ITGA2	Fw GTAGTGTGCTGTGTTCAGTTG<br>Rev GTCTGAAAGAGCTGCAATTGC
ITGAV	Fw GCTGTCGGAGATTTCAATGGT<br>Rev TCTGCTCGCCAGTAAAATTGT
ITGA6	Fw ATGGAGAAGTTGGAGGTGCAG<br>Rev CAAGTCATCATACGGAGCTCC
ITGB1	Fw ACAATGGAGAGTGCGTCTGC<br>Rev CCATTGCAGATCTGTCCGTTG
ITGB3	Fw AGGATGATCTGTGGAGCATCC<br>Rev ATTGAAGCGGGTCACCTGGT
ITGB4	Fw CAGTTCTGCGAGTATGACAAC<br>Rev ATTGCTGTCGATGCAGGTGG
MMP14	Fw TCCAGCAACTTTATGGGGGT<br>Rev TTCCCGTCACAGATGTTGGG

PSD4	Fw AAGCATTGACCTGGGAATCAGG<br>Rev TCCTGGGAGTTAAGAGTCACC
ARF6	Fw ATGGGGAAGGTGCTATCCAAAATC<br>Rev GCAGTCCACTACGAAGATGAGACC
FOXA1	Fw CAGGGCTCCATGGTTGTATTG<br>Rev GTGTTCATGGAGTTCATGGAGC
SNAI1	Fw CACTATGCCGCGCTCTTTC<br>Rev<br>GCTGGAAGGTAAACTCTGGATTAGA
TGFBR1	Fw ACGGCGTTACAGTGTTCTG<br>Rev GGTGTGGCAGATATAGACC
**Gene-specific ChIP primers**	**Sequence**
PSD4, Set 1	Fw TGGCAGGTTCTTTCTAGGCT<br>Rev CTCCTCCTCACCACAACACA
PSD4, Set 2	Fw GTGTGAGGAGGGATTGAGGT<br>Rev CCCAGGTTCTCAGTCTTGCT
ARF6, Set 1	Fw CCCAGATCCAGGCCATACAA<br>Rev GAAGAGTTCCTGTGCCTTGC
ARF6, Set 2	Fw AAACATGGGCTGGTCTTGAC<br>Rev GTGTCTCGTGTGGAATCTGC

SUPPLEMENTARY FIGURE TITLES AND LEGENDS

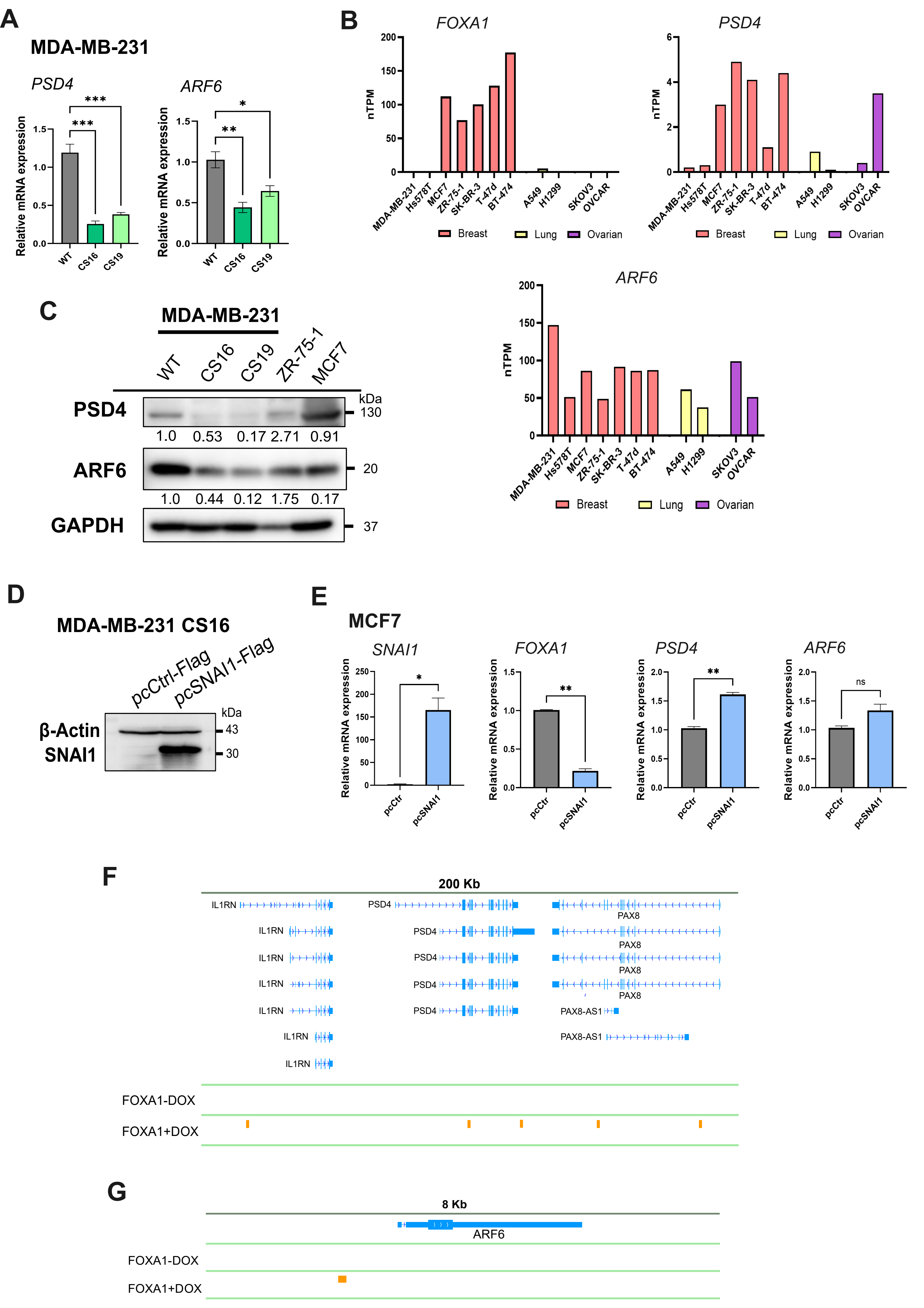

A
MDA-MB-231
PSD4
ARF6
Relative mRNA expression
WT
CS16
CS19
B
FOXA1
PSD4
ARF6
nTPM
MDA-MB-231
Hs578T
MCF7
ZR-75-1
SK-BR-3
T-47d
BT-474
A549
H1299
SKOV3
OVCAR
Breast
Lung
Ovarian
C
MDA-MB-231
WT
CS16
CS19
ZR-75-1
MCF7
PSD4
kDa
130
1.0 0.53 0.17 2.71 0.91
ARF6
20
1.0 0.44 0.12 1.75 0.17
GAPDH
37
D
MDA-MB-231 CS16
pcCtrl-Flag
pcSNAI1-Flag
kDa
β-Actin
43
SNAI1
30
E
MCF7
SNAI1
FOXA1
PSD4
ARF6
Relative mRNA expression
pcCtr
pcSNAI1
ns
F
200 Kb
IL1RN
PSD4
PAX8
PAX8-AS1
FOXA1-DOX
FOXA1+DOX
G
8 Kb
ARF6
FOXA1-DOX
FOXA1+DOX

Suppl. Fig.1

Supplementary Fig. 1. *SNAI1* regulates *PSD4*, *ARF6* and *FOXA1* expression

A RT-qPCR analysis of *PSD4* and *ARF6* mRNA levels in MDA-MB-231 WT and *SNAI1*-KO cells. Values represent fold-change of mRNA expression normalized to *GAPDH* and expressed relative to the level in MDA-MB-231 WT cells. Data are presented as mean values of three biological replicates ± SEM, each in technical triplicates and p-values (*$p \leq 0.05$, **$p \leq 0.01$, ***$p \leq 0.001$) are based on one-way ANOVA, followed by multiple paired comparisons conducted by means of Tukey's post-test method. **B** Expression levels (as normalized transcripts per million, nTPM) of the three indicated genes across specific cancer cell lines. The bars in the graphs are colored according to organ type. The data are derived from the human protein atlas database (www.proteinatlas.org). **C** Protein expression levels of PSD4 and ARF6 in MDA-MB-231 WT and *SNAI1*-KO cells and average densitometric analysis. GAPDH serves as loading control. Immunoblots are representative of at least two biological replicates along with molecular mass markers in kDa. **D** Representative immunoblot of at least two biological replicates along with molecular mass markers in kDa showing protein expression levels of FLAG-tagged SNAI1 after transient transfection of MDA-MB-231 *SNAI1*-KO cells with pcCtrl-FLAG vector or pcSNAI1-FLAG. β-Actin serves as a loading control. **E** RT-qPCR analysis of *SNAI1*, *FOXA1*, *PSD4* and *ARF6* mRNA levels in MCF7 cells transiently transfected with pcCtrl or pcSNAI1-FLAG (pcSNAI1). Values represent fold-change of mRNA expression normalized to *GAPDH* and expressed relative to the level in MCF7 cells transfected with pcCtrl. Data in E are presented as mean values of three biological replicates ± SEM, each in technical triplicates and p-values (*$p \leq 0.05$; **$p \leq 0.01$; ns, not significant) are based on *t*-test with Welch's correction. **F, G** FOXA1 binding to the *PSD4* (**F**) and *ARF6* (**G**) genomic regions upon FOXA1 overexpression (+Dox) and in the absence of FOXA1 (-Dox) in MCF-7 BRCA cells. The five *PSD4* (flanking genes) and one

ARF6 transcript isoforms are shown in blue, while the orange lines depict the binding sites of FOXA1 to *PSD4* and *ARF6*. All uncropped (original) immunoblots are shown as a supplemental file.

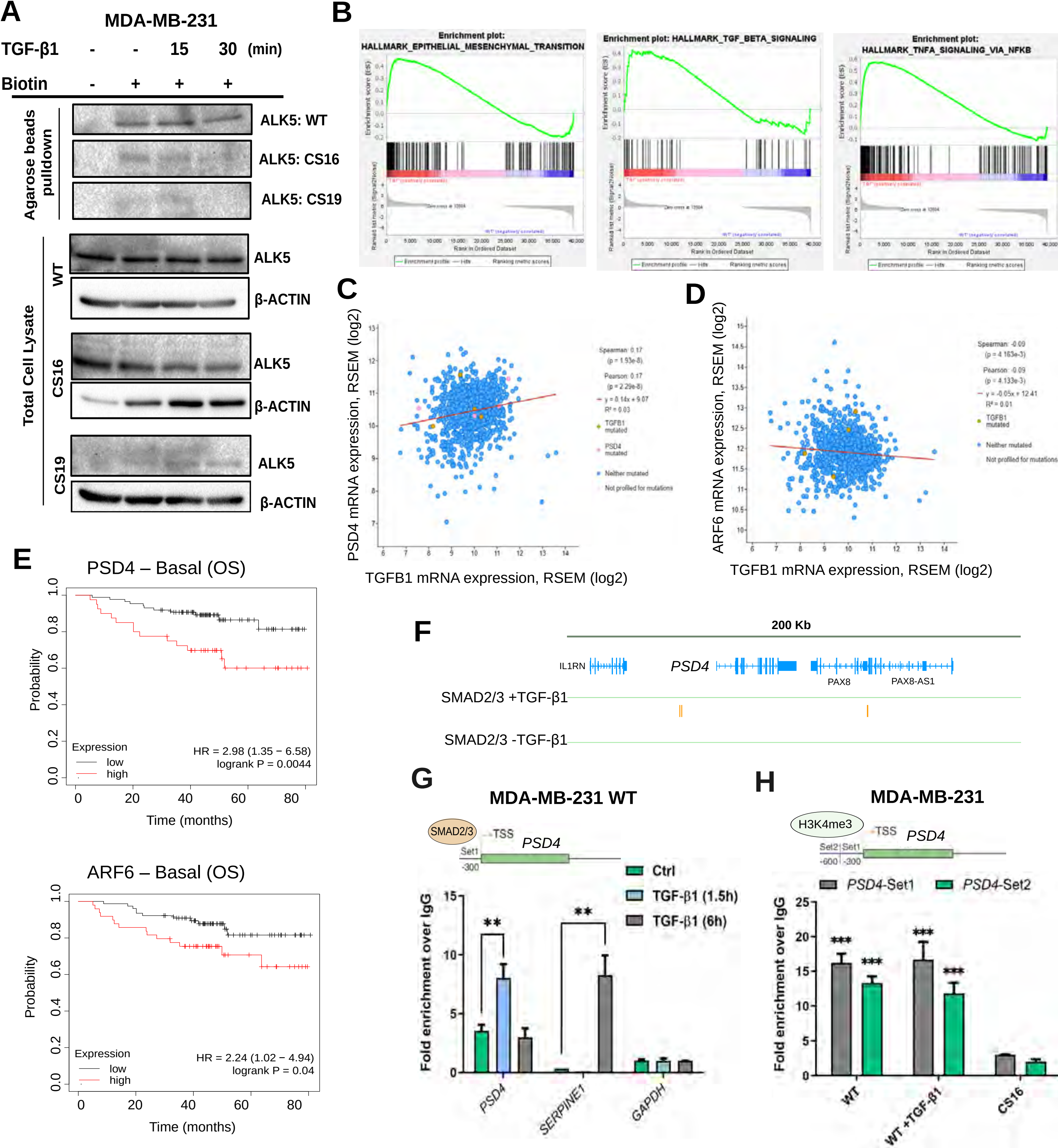
A
MDA-MB-231
TGF-β1 - - 15 30 (min)
Biotin - + + +
Agarose beads pulldown
ALK5: WT
ALK5: CS16
ALK5: CS19
Total Cell Lysate
WT
ALK5
β-ACTIN
CS16
ALK5
β-ACTIN
CS19
ALK5
β-ACTIN
B
Enrichment plot: HALLMARK_EPITHELIAL_MESENCHYMAL_TRANSITION
Enrichment plot: HALLMARK_TGF_BETA_SIGNALING
Enrichment plot: HALLMARK_TNFA_SIGNALING_VIA_NFKB
C
PSD4 mRNA expression, RSEM (log2)
TGFB1 mRNA expression, RSEM (log2)
D
ARF6 mRNA expression, RSEM (log2)
TGFB1 mRNA expression, RSEM (log2)
E
PSD4 – Basal (OS)
Probability
Time (months)
Expression
low
high
HR = 2.98 (1.35 − 6.58)
logrank P = 0.0044
ARF6 – Basal (OS)
Probability
Time (months)
Expression
low
high
HR = 2.24 (1.02 − 4.94)
logrank P = 0.04
F
200 Kb
IL1RN
PSD4
PAX8
PAX8-AS1
SMAD2/3 +TGF-β1
SMAD2/3 -TGF-β1
G
MDA-MB-231 WT
SMAD2/3
TSS
PSD4
Set1
-300
Ctrl
TGF-β1 (1.5h)
TGF-β1 (6h)
Fold enrichment over IgG
PSD4
SERPINE1
GAPDH
H
MDA-MB-231
H3K4me3
TSS
PSD4
Set2
Set1
-600
-300
PSD4-Set1
PSD4-Set2
Fold enrichment over IgG
WT
WT +TGF-β1
CS16

Suppl. Fig.2

Supplementary Fig. 2. SNAI1 and TGF-β signaling analysis

A Representative pulldown assay followed by immunoblotting of three biological replicates, along with molecular mass markers in kDa, showing cell surface biotinylated ALK5 protein levels in MDA-MB-231 WT and *SNAI1*-KO cells. The corresponding total cell lysates are also analyzed along with β-actin that serves as a loading control. **B** Enrichment plots of the significantly enriched signatures of EMT, TGF-β and TNFα signaling in MDA-MB-231 WT cells stimulated with TGF-β. **C, D** Scatter plots demonstrating a positive correlation between *TGFB1* and *PSD4* (**C**) or *ARF6* (**D**) mRNA expression, respectively, in TNBC/basal breast cancer samples obtained from TCGA datasets. **E** Higher PSD4 and ARF6 levels correlate with shorter overall survival (OS, n=126) in human basal BRCA patients. Basal BRCA samples were stratified and compared in the KM plotter database using autoselect cut-off. **F** SMAD2/3 binding to the *PSD4* genomic regions upon TGFβ stimulation (+TGF-β1) and in the absence of TGFβ (-TGF-β1) in MCF10A-II cells. The *PSD4* transcript is shown in blue, while the orange lines depict the binding sites of SMAD2/3 on the *PSD4* gene. **G** ChIP-qPCR analysis of SMAD2/3 binding upstream of the *PSD4* transcription starting site of MDA-MB-231 WT cells after stimulation or not with 5 ng/ml TGF-β1 for 1.5 and 6 h. Binding upstream the *SERPINE1* gene is used as a positive control and binding over *GAPDH* is used as a negative control. **H** ChIP-qPCR analysis of $H3K4me^3$ occupancy upstream of the *PSD4* transcription starting site of MDA-MB-231 WT cells after stimulation or not with 5 ng/ml TGF-β1 for 1.5 h and in CS16 *SNAI1*-KO cells. Values in panels (**G**) and (**H**) represent fold-enrichment over IgG and the data are presented as mean values of three biological replicates ± SEM, each in technical triplicates and p-values (**$p \leq 0.01$; ***$p \leq 0.001$) are based on two-way ANOVA, followed by multiple paired comparisons conducted by means of Tukey's post-test method.

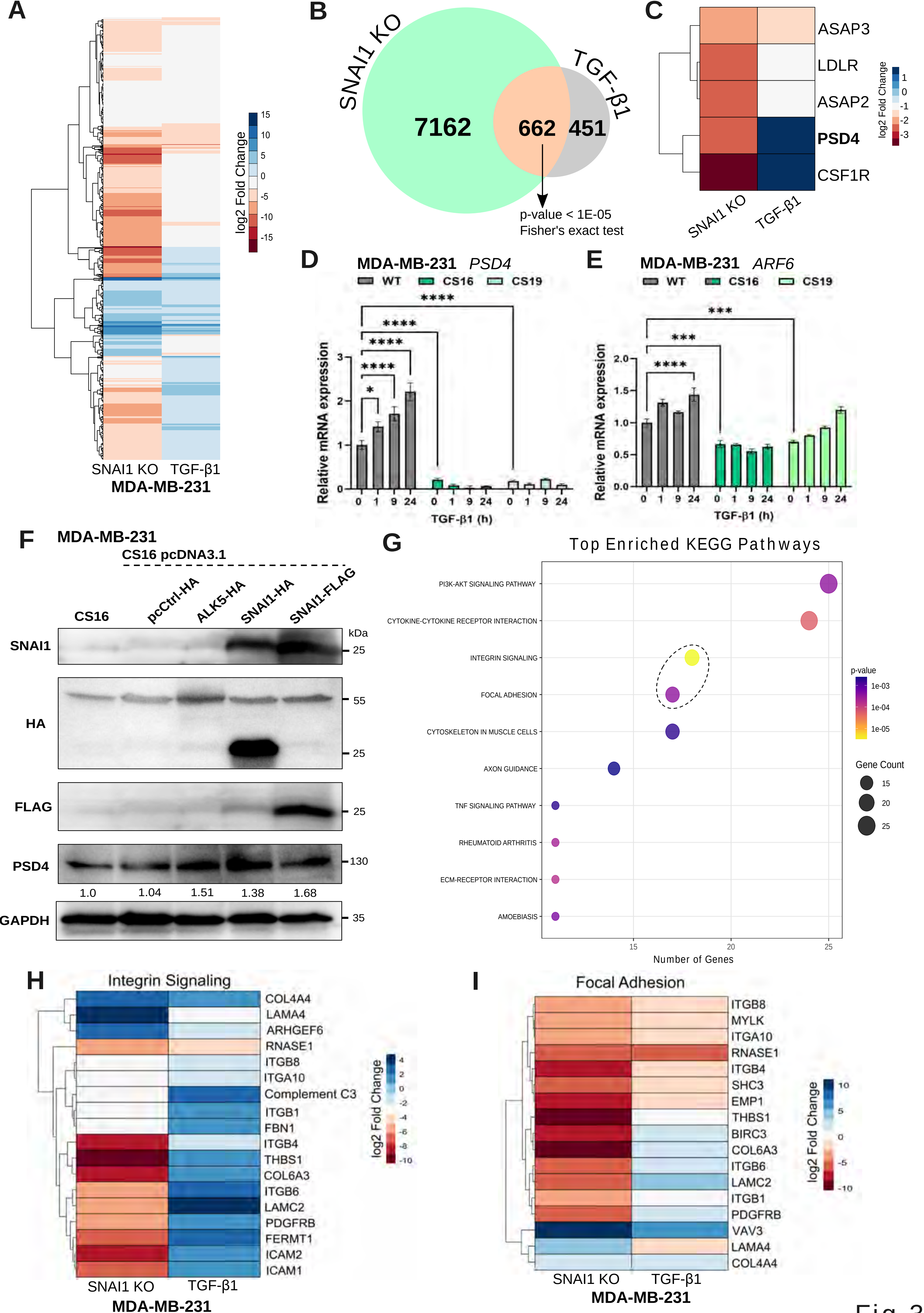

A
SNAI1 KO
TGF-β1
MDA-MB-231
log2 Fold Change
B
SNAI1 KO
TGF-β1
7162
662
451
p-value < 1E-05
Fisher's exact test
C
ASAP3
LDLR
ASAP2
PSD4
CSF1R
SNAI1 KO
TGF-β1
D
MDA-MB-231 PSD4
WT CS16 CS19
Relative mRNA expression
TGF-β1 (h)
E
MDA-MB-231 ARF6
WT CS16 CS19
Relative mRNA expression
TGF-β1 (h)
F
MDA-MB-231
CS16 pcDNA3.1
CS16
pcCtrl-HA
ALK5-HA
SNAI1-HA
SNAI1-FLAG
kDa
SNAI1
HA
FLAG
PSD4
GAPDH
1.0
1.04
1.51
1.38
1.68
G
Top Enriched KEGG Pathways
PI3K-AKT SIGNALING PATHWAY
CYTOKINE-CYTOKINE RECEPTOR INTERACTION
INTEGRIN SIGNALING
FOCAL ADHESION
CYTOSKELETON IN MUSCLE CELLS
AXON GUIDANCE
TNF SIGNALING PATHWAY
RHEUMATOID ARTHRITIS
ECM-RECEPTOR INTERACTION
AMOEBIASIS
Number of Genes
p-value
Gene Count
H
Integrin Signaling
COL4A4
LAMA4
ARHGEF6
RNASE1
ITGB8
ITGA10
Complement C3
ITGB1
FBN1
ITGB4
THBS1
COL6A3
ITGB6
LAMC2
PDGFRB
FERMT1
ICAM2
ICAM1
SNAI1 KO
TGF-β1
MDA-MB-231
I
Focal Adhesion
ITGB8
MYLK
ITGA10
RNASE1
ITGB4
SHC3
EMP1
THBS1
BIRC3
COL6A3
ITGB6
LAMC2
ITGB1
PDGFRB
VAV3
LAMA4
COL4A4
SNAI1 KO
TGF-β1
MDA-MB-231

Fig.3

Supplementary Fig. 3. SNAI1 regulates integrin family genes and adhesion to matrix

A, B RT-qPCR analysis of *ITGAV*, *ITGB1*, *ITGB3* and *ITGB5* mRNA levels in ZR-57-1 (**A**) and MCF7 (**B**) cells upon transient transfection with pcCtrl or pcSNAI1-FLAG (pcSNAI). Values represent fold-change of mRNA expression normalized to *GAPDH* and presented relative to the pcCtrl condition. **C** RT-qPCR analysis of *ITGA2*, *ITGAV*, *ITGB1* and *ITGB3* mRNA levels in MDA-MB-231 WT cells upon treatment with 5 ng/ml TGF-β1 for the indicated time periods. Values represent fold-change of mRNA expression normalized to *GAPDH*. Data in A and B are presented as mean values of three biological replicates ± SEM, each in technical triplicates and p-values (*p ≤ 0.05; **p ≤ 0.01; ns, not significant) are shown based on *t*-test with Welch's correction. Data in C are presented as mean values of three biological replicates ± SEM, each in technical triplicates and p-values (*p ≤ 0.05; ns, not significant) are based on one-way ANOVA, followed by multiple paired comparisons conducted by means of Tukey’s post-test method. **D, E** Extracellular matrix-mediated cell adhesion assay of collagen type I or fibronectin with ZR-57-1 (**D**) or MCF7 (**E**) cells after quantification. **F** Representative immunofluorescence images of integrin β1 (green), using the monoclonal P5D4 antibody used for adhesion blocking experiments and direct fluorescence of F-actin (magenta, phalloidin) in MDA-MB-231 WT cells. Nuclei are stained in blue with DAPI. Scale bars, 50 µm. **G** Quantification of extracellular matrix-mediated cell adhesion assay in MDA-MB-231 WT cells after incubation of live cells with anti-integrin P5D2 monoclonal antibody prior to the adhesion assay. Data in D, E and G are presented as mean values of three biological replicates ± SEM, each in technical triplicates and p-values are based two-way ANOVA, followed by multiple paired comparisons conducted by means of Tukey’s post-test method. p-values: *p ≤ 0.05; **p ≤ 0.01; ***p ≤ 0.001; **** p ≤ 0.0001; ns, not significant. **H** Representative immunoblot of at least two biological replicates along with molecular mass

markers in kDa showing protein expression levels of FAK and pFAK in MDA-MB-231 WT and *SNAI1*-KO cells upon treatment with 5 ng/ml TGF-β1 for the indicated time periods and average densitometric analysis. ALIX serves as loading control.

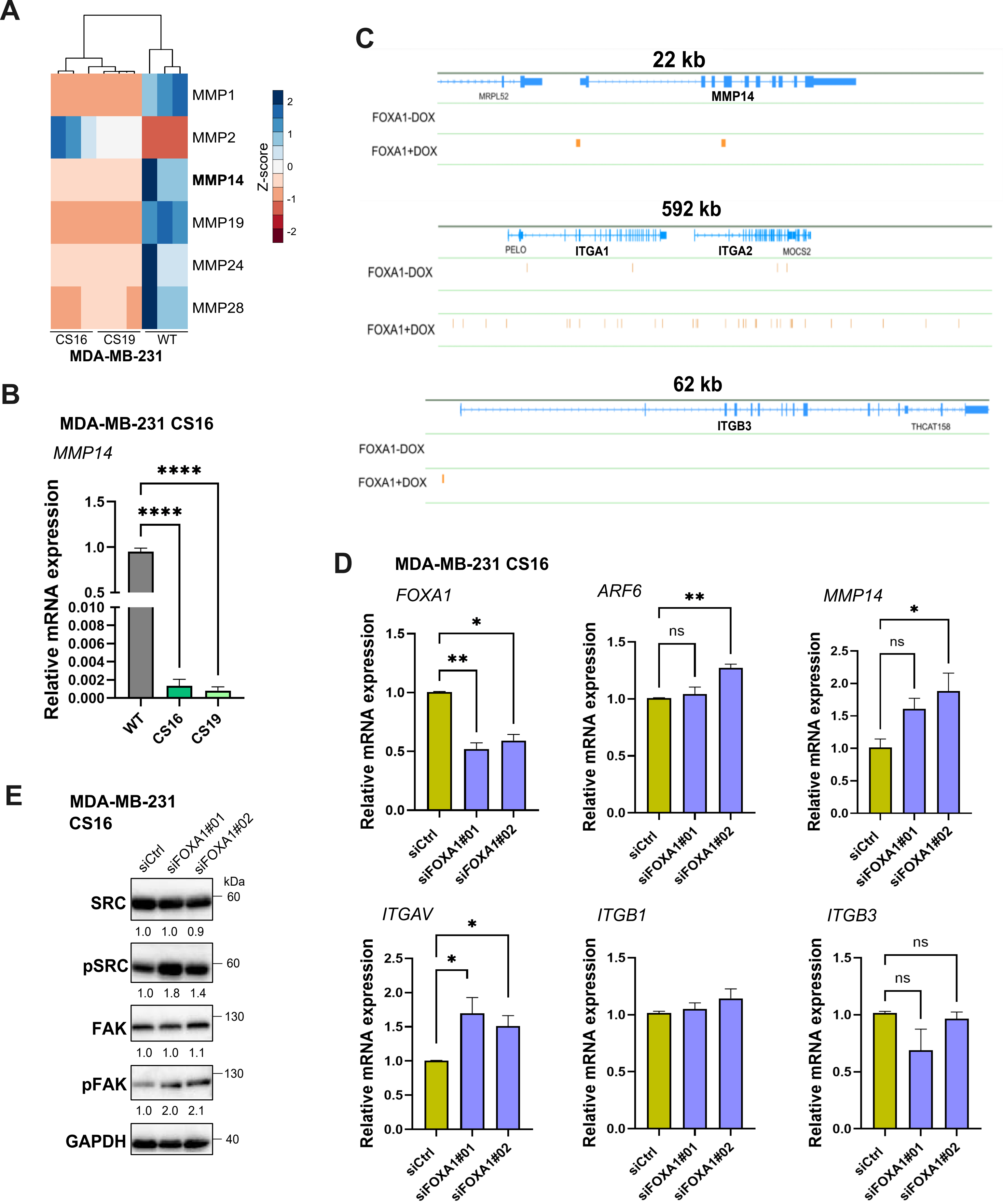

A
MMP1
MMP2
MMP14
MMP19
MMP24
MMP28
Z-score
2
1
0
-1
-2
CS16
CS19
WT
MDA-MB-231
B
MDA-MB-231 CS16
MMP14
Relative mRNA expression
****
****
WT
CS16
CS19
C
22 kb
MRPL52
MMP14
FOXA1-DOX
FOXA1+DOX
592 kb
PELO
ITGA1
ITGA2
MOCS2
62 kb
ITGB3
THCAT158
D
MDA-MB-231 CS16
FOXA1
ARF6
MMP14
ITGAV
ITGB1
ITGB3
siCtrl
siFOXA1#01
siFOXA1#02
ns
E
MDA-MB-231 CS16
kDa
SRC
pSRC
FAK
pFAK
GAPDH
1.0 1.0 0.9
1.0 1.8 1.4
1.0 1.0 1.1
1.0 2.0 2.1

Suppl. Fig.4

Supplementary Fig. 4. SNAI1 and FOXA1 regulate integrin and MMP family genes

A Heat map of the indicated six MMP family members in wild-type (WT) and *SNAI1* knockout (CS16 and CS19) cells. The color-coded Z-score of the normalized expression value of statistical significance is explained. **B** RT-qPCR analysis of *MMP14* mRNA levels in MDA-MB-231 WT and *SNAI1*-KO cells. Values represent fold-change of mRNA expression normalized to *GAPDH* and expressed relative to the level in MDA-MB-231 WT cells. **C** FOXA1 binding to the *MMP14*, *ITGA1*, *ITGA2* and *ITGB3* genomic regions upon FOXA1 overexpression (+Dox) and in the absence of FOXA1 (-Dox) in MCF-7 BRCA cells. The specific and some flanking genes are shown in blue, while the orange lines depict the binding sites of FOXA1 to *PSD4* and *ARF6*. **D** RT-qPCR analysis of the mRNA levels of the six indicated genes in *SNAI1*-KO cells upon FOXA1 knockdown. Values represent fold-change of mRNA expression normalized to *GAPDH* and expressed relative to the level in siCtrl cells. **E** Representative immunoblot and densitometric analysis of at least two independent biological replicates along with molecular mass markers in kDa showing protein expression levels of SRC, pSRC, FAK and pFAK in CS16 *SNAI1*-KO cells upon FOXA1 knockdown with two independent siRNAs. GAPDH serves as a loading control. Data in B are presented as mean values of three biological replicates ± SEM, each in technical triplicates and p-values (****$p \leq 0.0001$) are based on one-way ANOVA, followed by multiple paired comparisons conducted by means of Tukey's post-test method. Data in D are presented as mean values of three biological replicates ± SEM, each in technical triplicates and p-values (*$p \leq 0.05$) are shown based on *t*-test with Welch's correction.

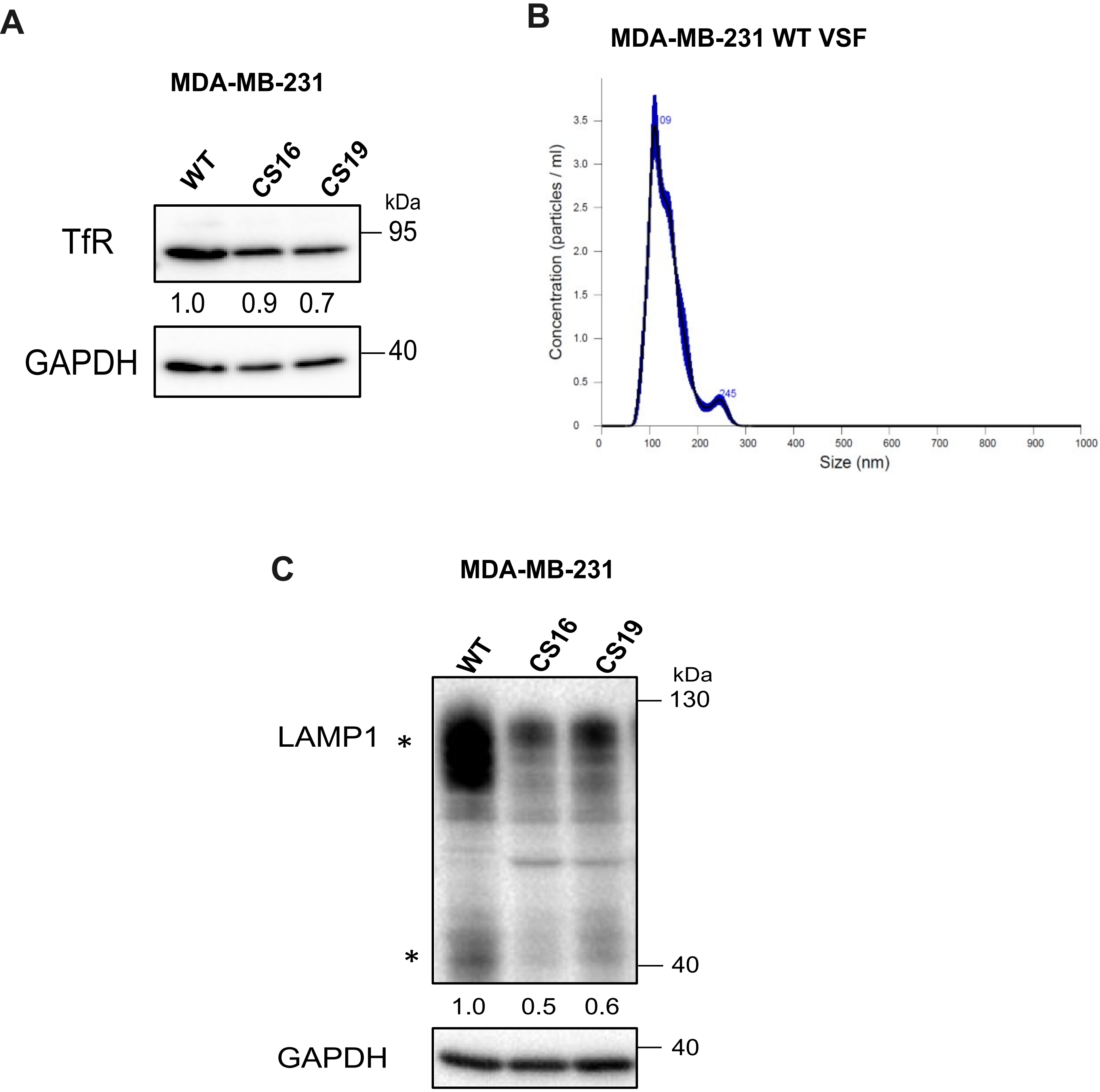

A
MDA-MB-231
WT
CS16
CS19
kDa
TfR
95
1.0
0.9
0.7
GAPDH
40
B
MDA-MB-231 WT VSF
Concentration (particles / ml)
Size (nm)
109
245
C
MDA-MB-231
WT
CS16
CS19
kDa
LAMP1
*
130
*
40
1.0
0.5
0.6
GAPDH
40

Suppl. Fig.5

Supplementary Fig. 5. SNAI1 knockout suppresses lysosomal maturation and extracellular vesicle release

A Representative immunoblot of three biological replicates along with molecular mass markers in kDa showing protein expression levels of transferrin receptor (TfR) in MDA-MB-231 WT and *SNAI1*-KO cells and average densitometric analysis. GAPDH serves as a loading control. **B** Representative NTA trace line indicating nanoparticle concentration released by MDA-MB-231 WT in the VSF that were labelled with PKH26, across nanoparticle size in nm. **C** Representative immunoblot of three biological replicates along with molecular mass markers in kDa showing protein expression levels of LAMP1 in MDA-MB-231 WT and *SNAI1*-KO cells and average densitometric analysis. GAPDH serves as a loading control and stars indicated different LAMP1 protein forms.

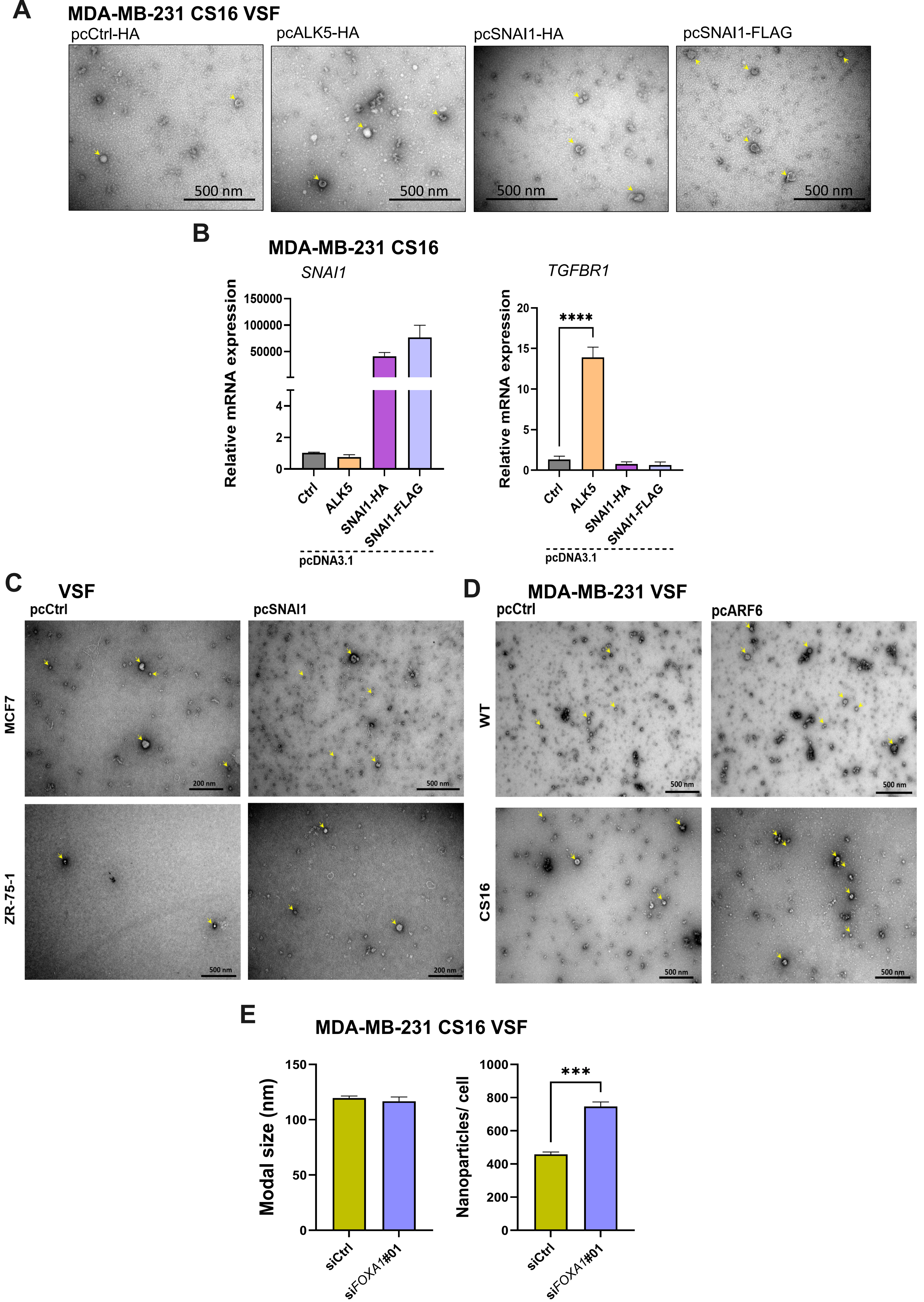

A
MDA-MB-231 CS16 VSF
pcCtrl-HA
pcALK5-HA
pcSNAI1-HA
pcSNAI1-FLAG
500 nm
B
MDA-MB-231 CS16
SNAI1
TGFBR1
Relative mRNA expression
150000
100000
50000
4
2
0
20
15
10
5
****
Ctrl
ALK5
SNAI1-HA
SNAI1-FLAG
pcDNA3.1
C
VSF
pcCtrl
pcSNAI1
MCF7
ZR-75-1
200 nm
500 nm
D
MDA-MB-231 VSF
pcCtrl
pcARF6
WT
CS16
500 nm
E
MDA-MB-231 CS16 VSF
Modal size (nm)
150
100
50
0
Nanoparticles/ cell
1000
800
600
400
200
0
***
siCtrl
siFOXA1#01

Suppl. Fig.6

Supplementary Fig. 6. Controls of EV analysis

A Representative TEM pictures depicting the morphology and the size of MDA-MB-231 CS16 *SNAI1*-KO derived particles after transient transfection of the cells with pcCtrl-HA vector, pcALK5-HA, pcSNAI1-HA or pcSNAI1-FLAG vectors (the latter two representing rescue experiments). Scale bars, 500 nm. **B** RT-qPCR analysis of *SNAI1* and *TGFBR1* mRNA levels in *SNAI1*-KO cells transiently transfected with vectors expressing nothing (Ctrl), the TGF-β type I receptor (TGFBR1) ALK5 or two epitope-tagged (HA- or FLAG-) forms of SNAI1 (rescue experiment). Values represent fold-change of mRNA expression normalized to *GAPDH* and expressed relative to the level in Ctrl cells. Data are presented as mean values of three biological replicates ± SEM, each in technical triplicates and p-values (****$p \leq 0.0001$) are based on one-way ANOVA, followed by multiple paired comparisons conducted by means of Tukey's post-test method. **C**, **D** Representative TEM pictures depicting the morphology and the size of MCF7 or ZR-57-1 cell derived particles after transient transfection of the cells with pcCtrl-FLAG vector or pcSNAI1-FLAG vectors (the latter representing rescue experiments) (**C**) and MDA-MB-231 WT cell derived particles after transient transfection of the cells with pcCtrl-GFP (pcCtrl) or pcARF6-GFP (pcARF6) vectors (**D**). Scale bars, 200 or 500 nm. **E** Nanoparticles released by CS16 *SNAI1*-KO cells upon *FOXA1* knockdown as quantified by NTA and reporting the size of the nanoparticles with the highest frequency (mode) within the sample and their normalized number to the total cell number. Data in I are presented as mean values of three biological replicates ± SEM, each in technical triplicates and p-values are based on two-way ANOVA, followed by multiple paired comparisons conducted by means of Tukey's post-test method. p-values: ***$p \leq 0.001$.
