# Supplementary material for: SNAI1 ablation alters integrin-mediated adhesion and endocytic fate
Source: Cell Death Dis. 2026 Aug 12;17(1):716. doi: 10.1038/s41419-026-09179-x (PMC13470233; doi:10.1038/s41419-026-09179-x)
Supplement: Supplementary file 2 [file 41419_2026_9179_MOESM2_ESM.pdf]

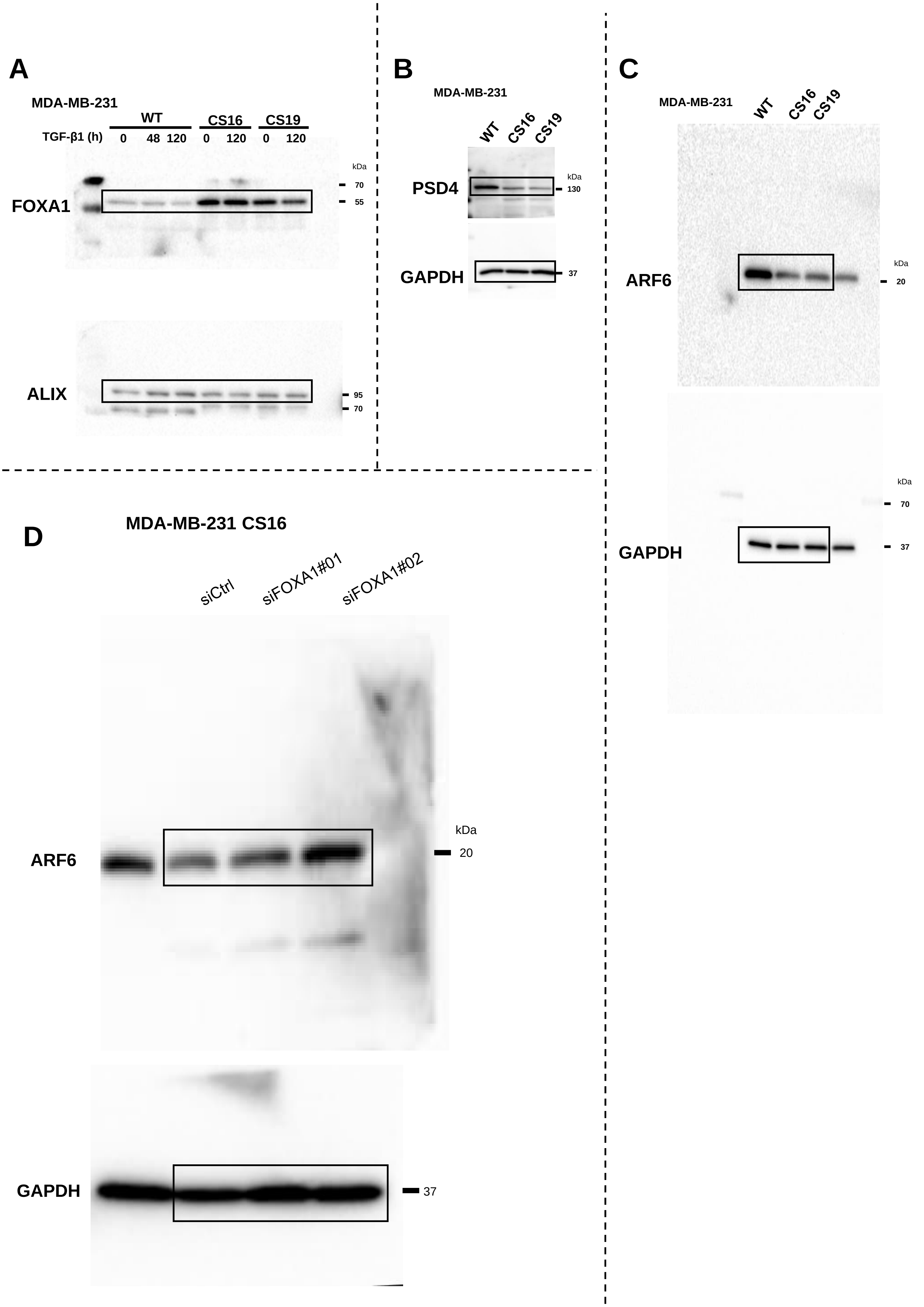
A
MDA-MB-231
WT
CS16
CS19
TGF-β1 (h)
0
48
120
0
120
0
120
kDa
70
55
FOXA1
ALIX
95
70
B
MDA-MB-231
WT
CS16
CS19
PSD4
kDa
130
GAPDH
37
C
MDA-MB-231
WT
CS16
CS19
kDa
ARF6
20
kDa
70
GAPDH
37
D
MDA-MB-231 CS16
siCtrl
siFOXA1#01
siFOXA1#02
kDa
ARF6
20
GAPDH
37

Figure 2

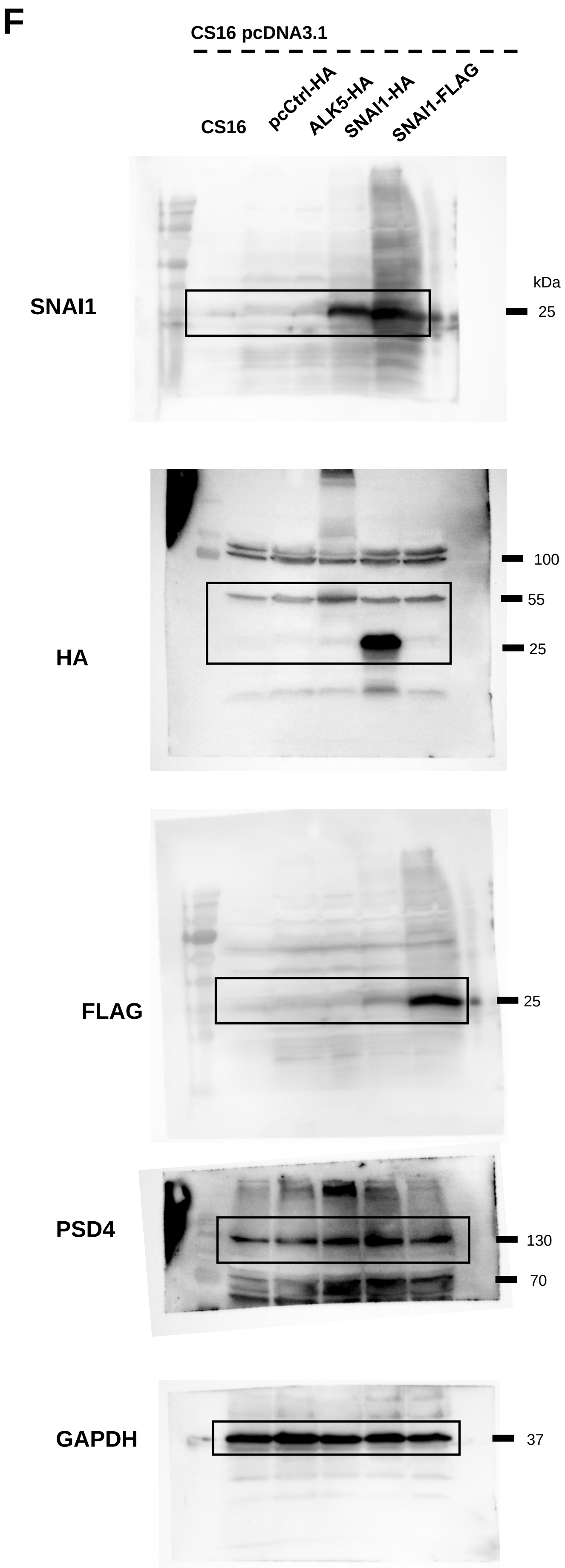
F
CS16 pcDNA3.1
CS16
pcCtrl-HA
ALK5-HA
SNAI1-HA
SNAI1-FLAG
SNAI1
kDa
25
HA
100
55
25
FLAG
25
PSD4
130
70
GAPDH
37

Figure 3

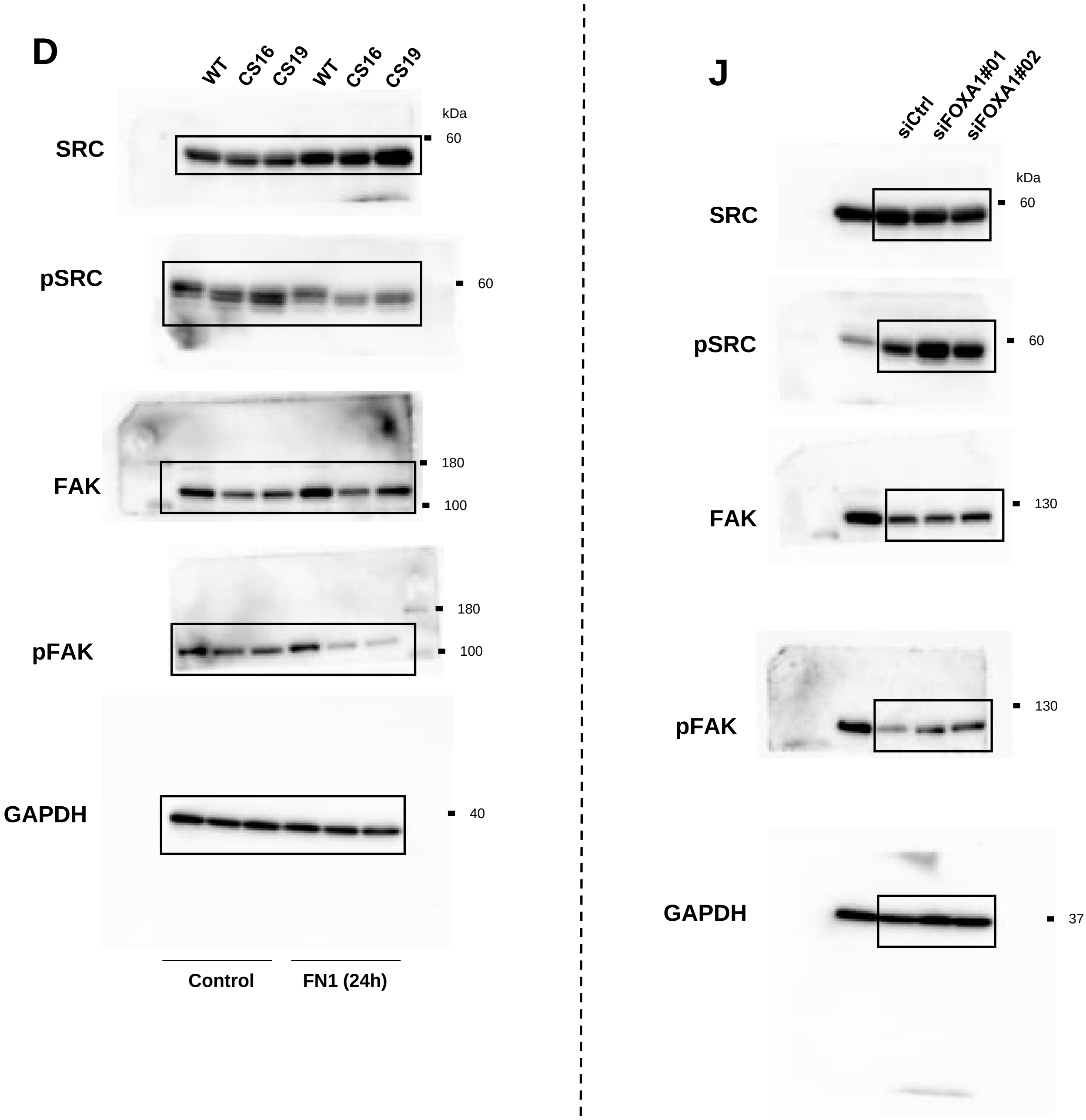
D
WT
CS16
CS19
WT
CS16
CS19
kDa
SRC
60
pSRC
60
FAK
180
100
pFAK
180
100
GAPDH
40
Control
FN1 (24h)
J
siCtrl
siFOXA1#01
siFOXA1#02
kDa
SRC
60
pSRC
60
FAK
130
pFAK
130
GAPDH
37

Figure 4

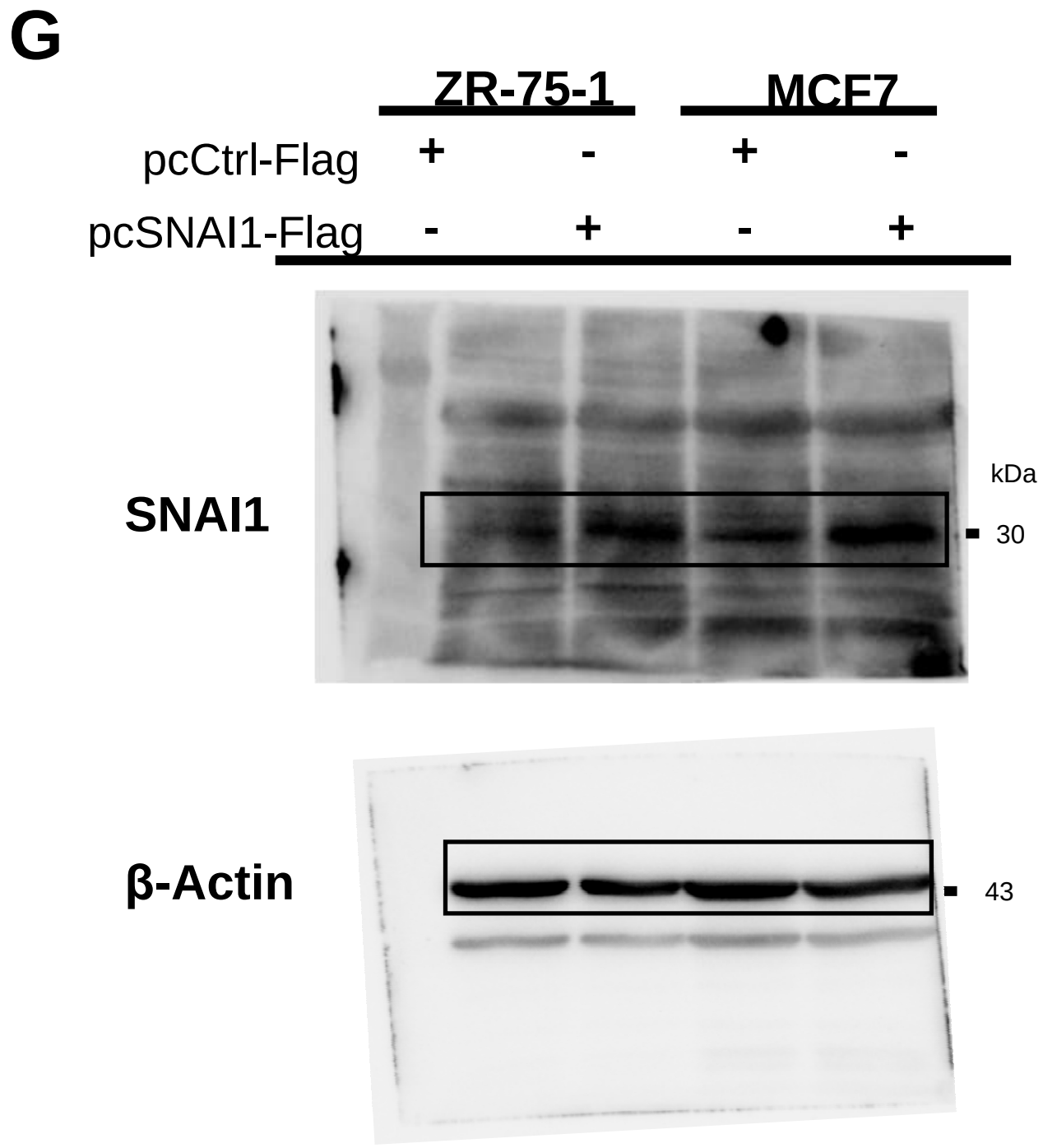

Figure 6

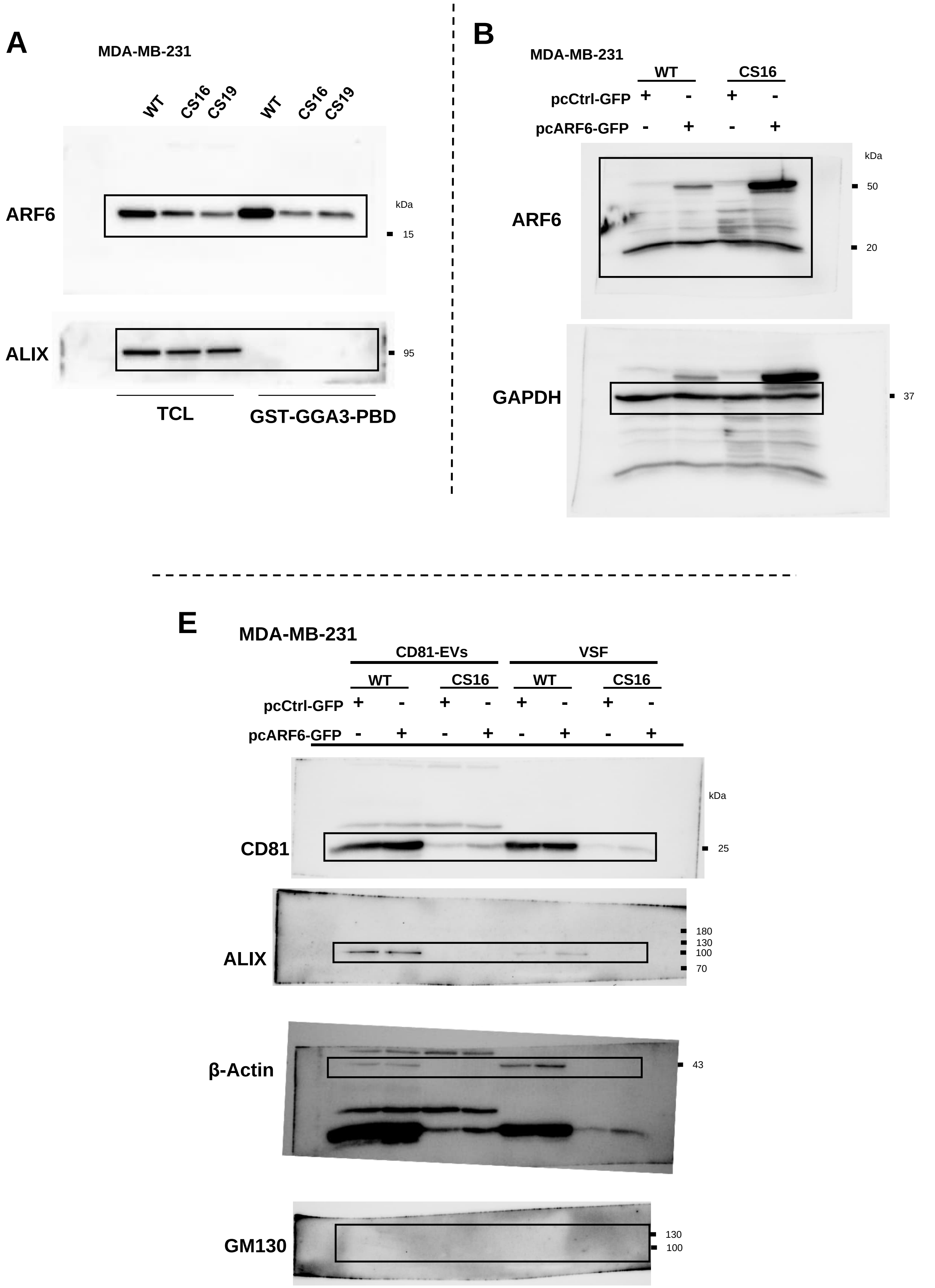
A
MDA-MB-231
WT
CS16
CS19
WT
CS16
CS19
ARF6
kDa
15
ALIX
95
TCL
GST-GGA3-PBD
B
MDA-MB-231
WT
CS16
pcCtrl-GFP + - + -
pcARF6-GFP - + - +
kDa
50
ARF6
20
GAPDH
37
E
MDA-MB-231
CD81-EVs
VSF
WT
CS16
WT
CS16
pcCtrl-GFP + - + - + - + -
pcARF6-GFP - + - + - + - +
kDa
CD81
25
ALIX
180
130
100
70
β-Actin
43
GM130
130
100

Figure 7

B

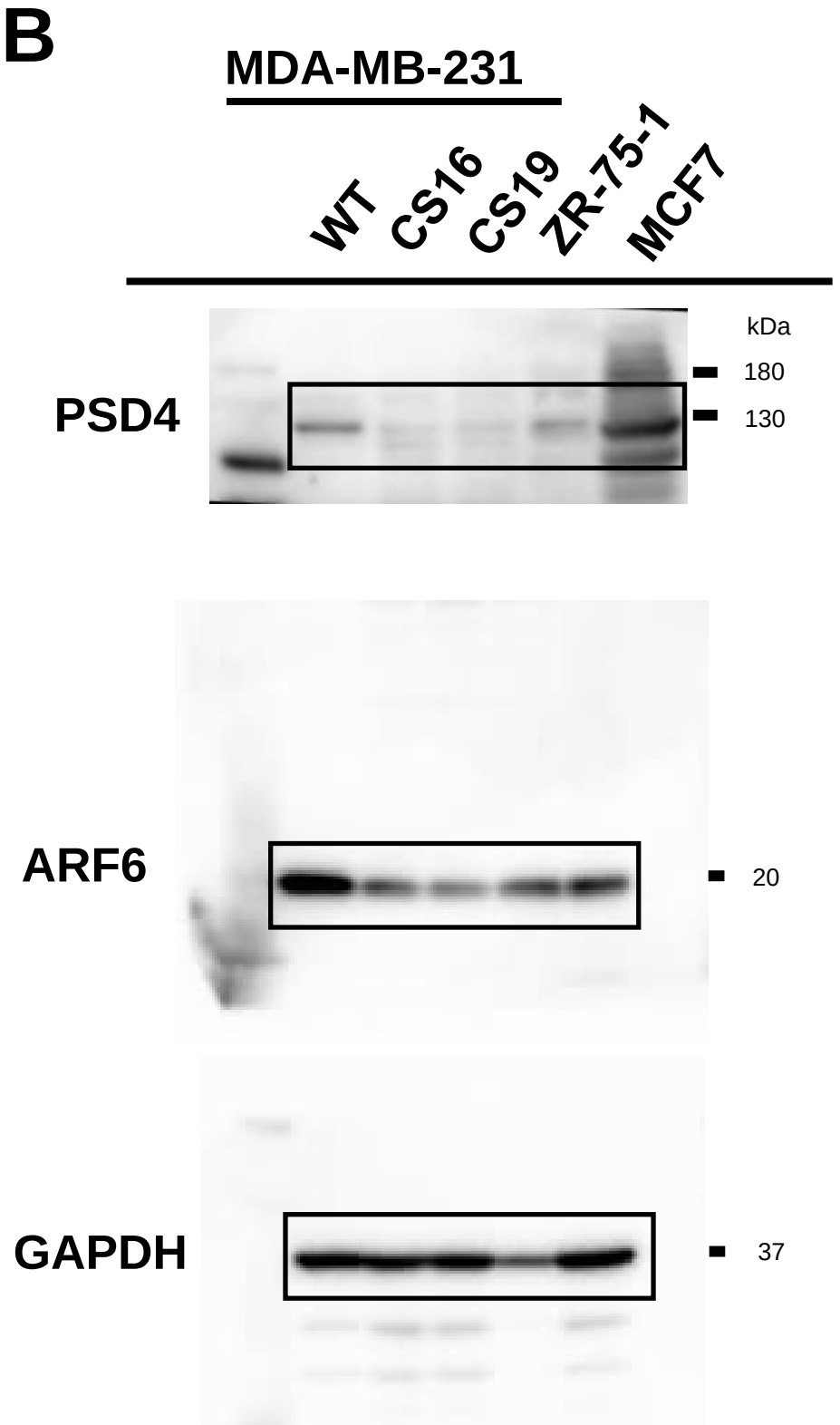
MDA-MB-231
WT
CS16
CS19
ZR-75-1
MCF7
PSD4
kDa
180
130
ARF6
20
GAPDH
37

C

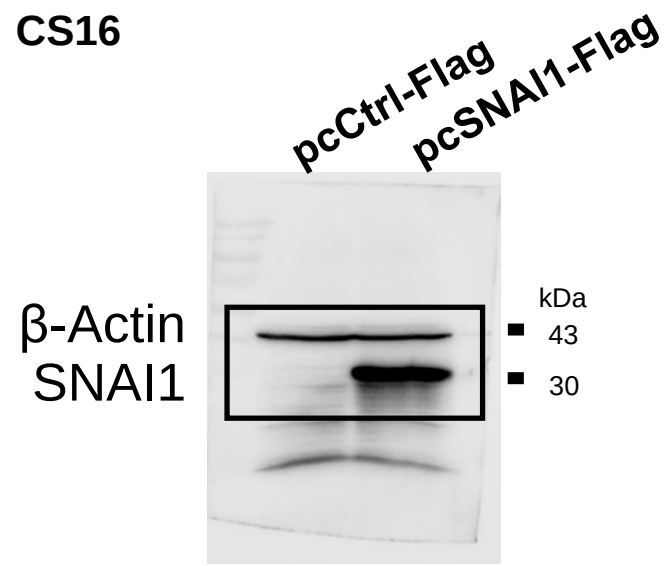
CS16
pcCtrl-Flag
pcSNAI1-Flag
β-Actin
SNAI1
kDa
43
30

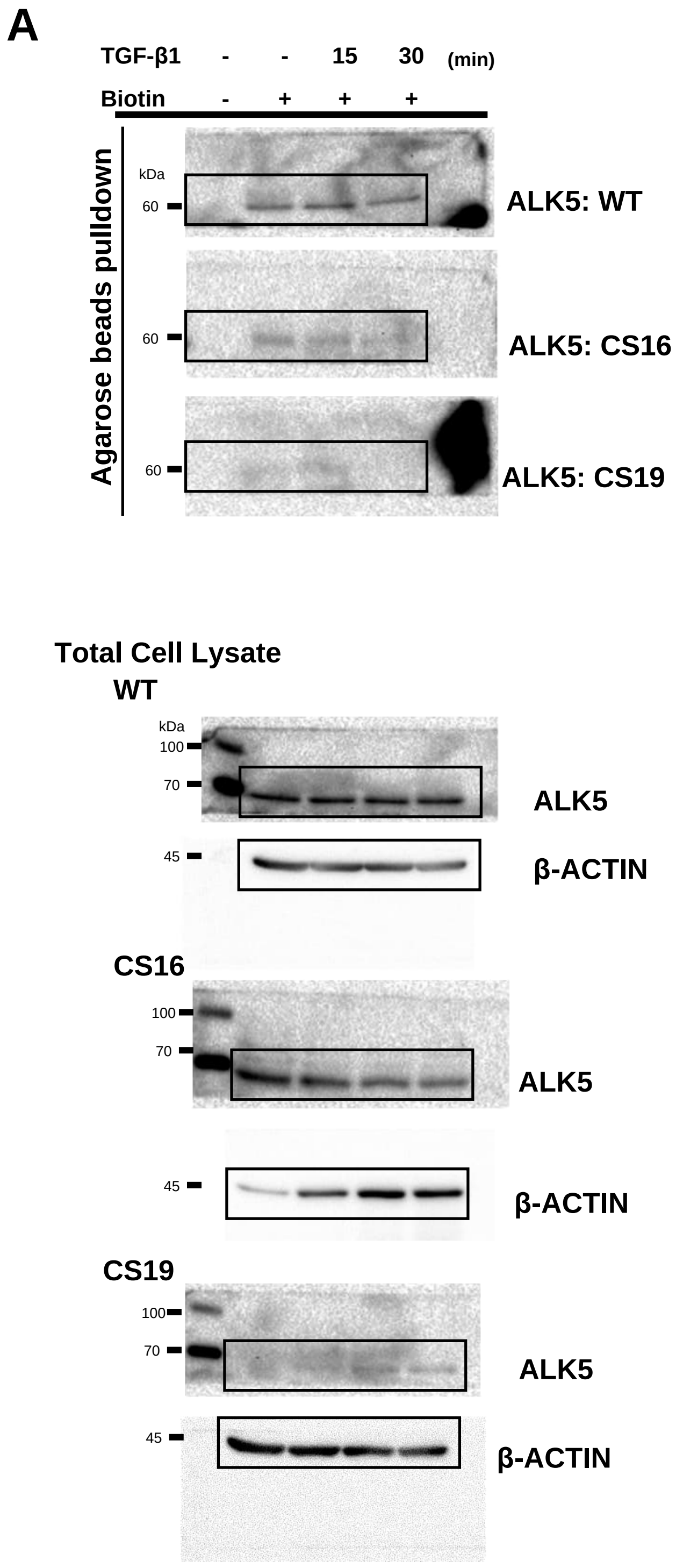
A
TGF-β1 - - 15 30 (min)
Biotin - + + +
Agarose beads pulldown
kDa
60
ALK5: WT
60
ALK5: CS16
60
ALK5: CS19
Total Cell Lysate
WT
kDa
100
70
ALK5
45
β-ACTIN
CS16
100
70
ALK5
45
β-ACTIN
CS19
100
70
ALK5
45
β-ACTIN

Suppl Fig 2

H

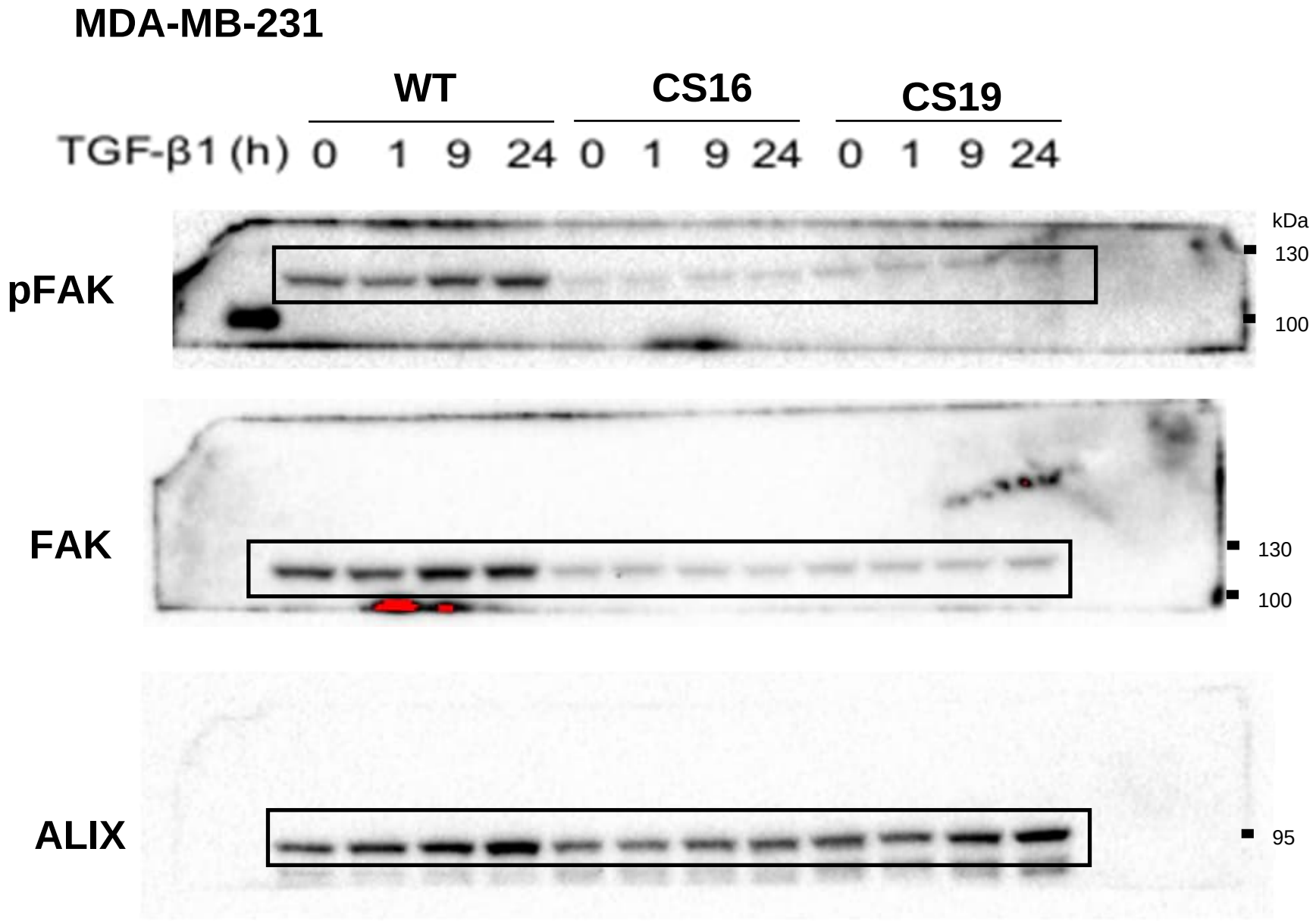
MDA-MB-231
WT
CS16
CS19
TGF-β1 (h) 0 1 9 24 0 1 9 24 0 1 9 24
pFAK
FAK
ALIX
kDa
130
100
130
100
95

A

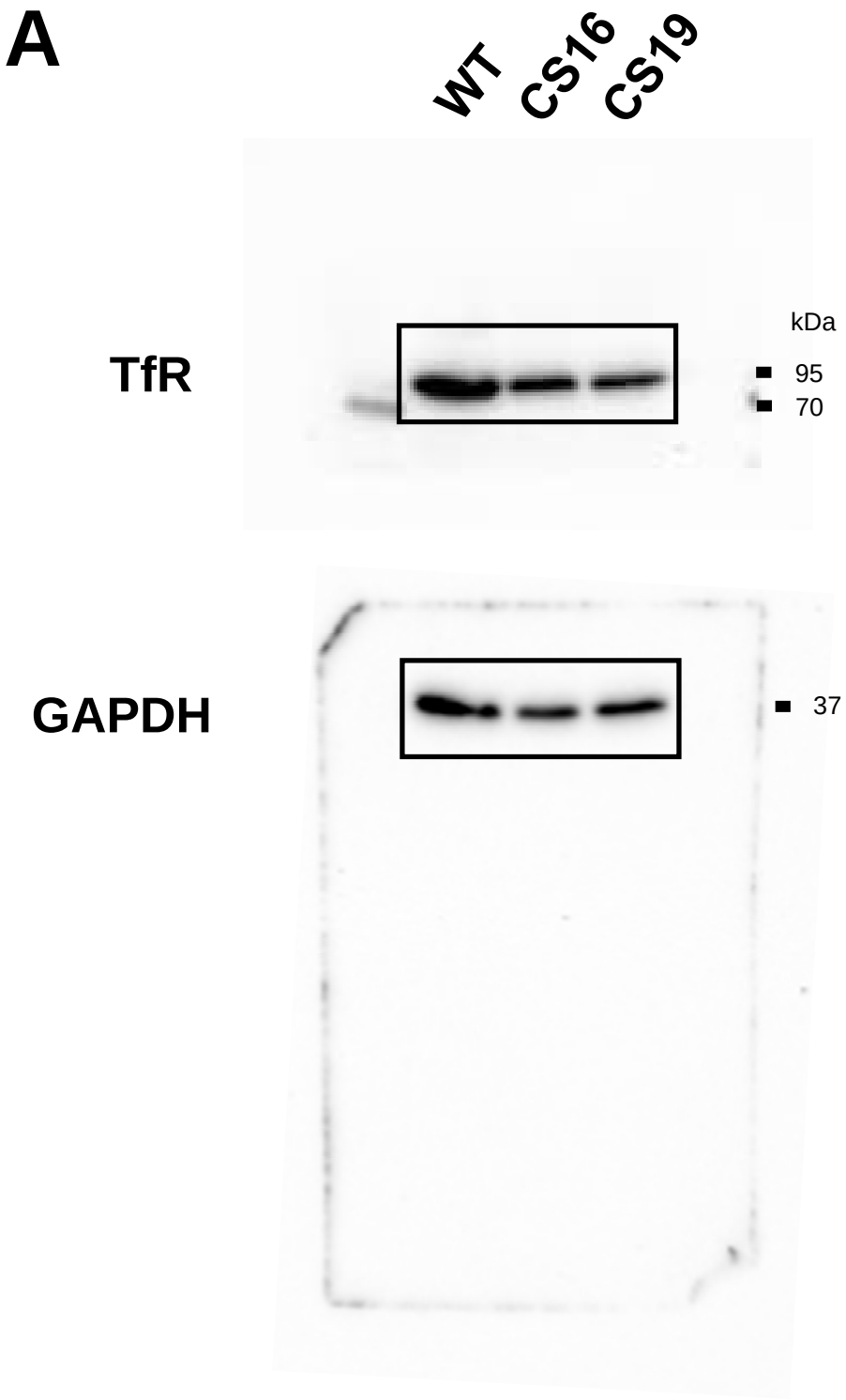

B

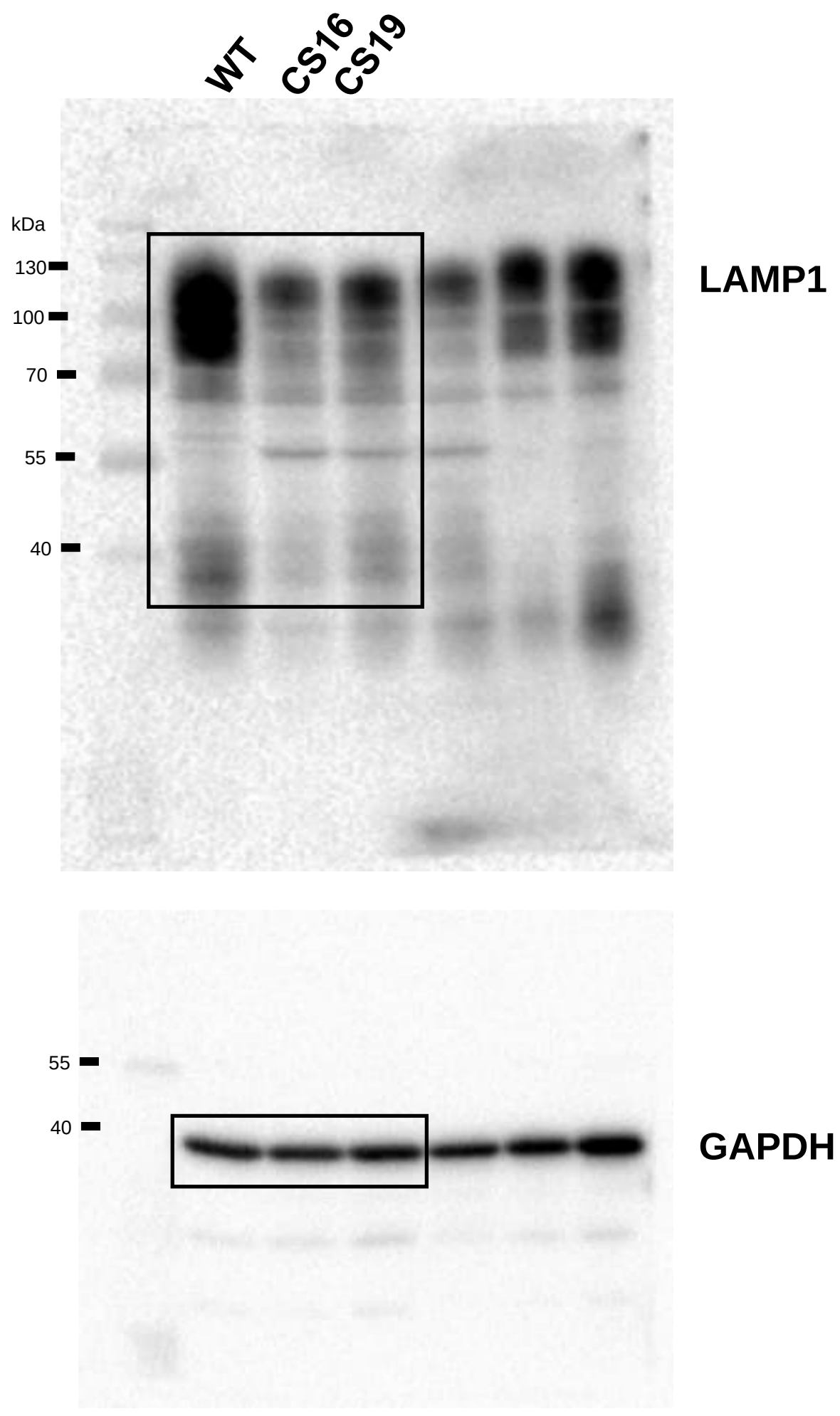

Suppl Fig 5

Original immunoblots.

A series of original immunoblots are collected, with rectangles marking the area of the immunoblot that was cropped and placed in the main or supplementary figures, along with reference to each of the corresponding figures.
